# Template‐Free Fabrication of Single Atom Fe‐Based Cathodes Unlock High‐Performing Anion‐Exchange Membrane Fuel Cells

**DOI:** 10.1002/advs.202501016

**Published:** 2025-07-17

**Authors:** John C. Douglin, Hideo Notsu, Shinsuke Nagata, Sapir Willdorf‐Cohen, Jinliu Zhong, Junya Ohyama, Jiawei Hu, Syeda M. Zahan, Andres O. Godoy, Changlai Wang, Oluwafemi Sanumi, Masayuki Tsushida, Karam Yassin, Jasna Jankovic, Charles E. Diesendruck, Yuta Nabae, Dario R. Dekel

**Affiliations:** ^1^ The Wolfson Department of Chemical Engineering Technion–Israel Institute of Technology Haifa 3200003 Israel; ^2^ Department of Materials Science and Engineering Institute of Science Tokyo 2‐12‐1 S8‐26, Ookayama Meguro‐ku Tokyo 152‐8552 Japan; ^3^ Faculty of Advanced Science and Technology Kumamoto University Kumamoto 860‐8555 Japan; ^4^ Center for Clean Energy Engineering University of Connecticut Storrs CT 06269 USA; ^5^ The Nancy & Stephen Grand Technion Energy Program (GTEP) Technion–Israel Institute of Technology Haifa 3200003 Israel; ^6^ Technical Division Kumamoto University 2‐39‐1 Kurokami, Chuo‐ku Kumamoto 860‐8555 Japan; ^7^ Schulich Faculty of Chemistry Technion─Israel Institute of Technology Haifa 3200003 Israel

**Keywords:** anion‐exchange membrane fuel cells, alkaline media, critical raw materials, high‐temperature, oxygen reduction reaction, single‐atom catalysts

## Abstract

Single‐atom catalysts (SACs) possessing well‐defined active sites of singular metal atoms have gained prominence in the field of electrocatalysis as they can be tuned to enhance activity and stability. In this study, a critical‐raw‐material (CRM)‐free SAC is synthesized for the oxygen reduction reaction (ORR) in alkaline media by pyrolyzing polyimide nanoparticles and Fe without using a sacrificial template. Upon purification, the resulting catalyst demonstrates outstanding performance as cathodes in anion‐exchange membrane fuel cells (AEMFCs) owing to sufficiently stabilized Fe single‐atoms at the FeN_4_ sites yielding a peak power density (*P*_max_) as high as ∼1.8 W cm^−2^ and specific power values up to 11.3 W ${\mathrm{mg}}_{\mathrm{PGM}}^{-1}$; the latter being the greatest reported among CRM‐free cathode AEMFCs. The SAC also shows remarkable in situ durability under a very high current density of 1000 mA cm^−2^, a first introduced here, with only a 2 mV h^−1^ decay. Most impressively, when the SAC is combined with a NiMo anode to test a completely CRM‐free high‐temperature (HT)‐AEMFC at 118 °C, a *P*_max_ of 372 mW cm^−2^ and limiting current density of ∼1.14 A cm^−2^ are achieved. This work represents a significant milestone in the development of durable SAC cathode catalysts for the next generation of CRM‐free AEMFCs.

## Graphical abstract

A high‐performance single‐atom critical‐raw‐material (CRM)‐free Fe‐based catalyst is designed using a template‐free manufacturing approach. This single‐atom catalyst (SAC) is incorporated into operando anion‐exchange membrane fuel cells using O_2_ and CO_2_‐free air displaying excellent performance. This SAC's adaptability is further demonstrated at high temperatures in ambient air by the utilization of CRM‐free materials in both electrodes.

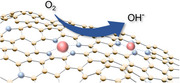

## 1 Introduction

Sustainable and renewable alternative energy technologies hold great promise as alternatives to fossil fuels for global energy security. Their versatile properties—existing abundantly in nature, derived from natural sources, or self‐replenishing—make them excellent candidates for a range of industries, including transportation, space and water heating, and energy generation.^[^ ^1^, ^2^ ^]^ In this respect, hydrogen‐powered polymer electrolyte fuel cells have demonstrated significant potential for providing an emissions‐free electric vehicle alternative to the transportation sector. Of these, the anion‐exchange membrane fuel cell (AEMFC) has shown the greatest potential to displace conventional proton exchange membrane fuel cells because of their alkaline operating environment, which allows for the employment of cost‐effective materials as catalysts.^[^ ^3^ ^]^ Since the first high‐performance AEMFCs were disclosed a decade ago,^[^ ^4^ ^]^ remarkable improvements in cell performance have been achieved, thanks to the developments in highly conducting anion‐exchange membranes (AEMs) and catalysts with improved activities toward the oxygen reduction reaction (ORR) in the alkaline environment.^[^ ^5^, ^6^, ^7^, ^8^, ^9^ ^]^

Despite this progress, the most critical challenges to overcome yet are cost and durability. State‐of‐the‐art AEMFCs still depend on large quantities of critical raw materials (CRMs) such as platinum group metal (PGM) catalysts (i.e. loadings of 0.6–1.4 mg_PGM_ cm^−2^), which are cost‐prohibitive due to diminishing globally available reserves and geopolitical issues that complicate availability.^[^ ^3^, ^10^ ^]^ This impingement continues to be a significant obstacle in the progression of AEMFCs as viable energy conversion devices within the transportation sector. Moreover, PGMs utilized in both electrodes have consistently shown the capability to meet the rigorous durability targets at high current densities.^[^ ^11^ ^]^ Consequently, a major priority has been to reduce PGM quantities used in AEMFCs without compromising performance and durability, to ultimately satisfy the criteria in both performance and durability established by the US Department of Energy (DOE).^[^ ^12^, ^13^, ^14^ ^]^

One strategy to reduce PGM quantities involves substituting them in one, or even both electrodes, with cheaper and more earth‐abundant‐based catalysts. While this approach has demonstrated promising AEMFC performance with PGM‐free catalysts in the cathode and can favorably impact cost, reported studies are limited to stability performances with relatively high PGM loadings in the anode and current densities ≤ 600 mA cm^−2^, or are completely devoid of stability performance. For instance, a report with a commercially available PGM‐free Fe‐N‐C catalyst coupled with a PGM‐based anode (loading of 0.6 mg_PtRu_ cm^−2^) delivered an outstanding peak power density (*P*_max_) of 2.1 W cm^−2^. After 150‐h at a constant current density of 600 mA cm^−2^, the voltage was found to have decreased by ∼10% overall.^[^ ^15^ ^]^ Similarly, a metal‐free nitrogen‐doped graphitic carbon catalyst combined with a PtRu anode with 0.4 mg_PtRu_ cm^−2^ delivered a *P*_max_ exceeding 0.9 W cm^−2^ and showed a voltage stability decay of 25% overall at a lower current density of 0.25 A cm^−2^ after 100‐h.^[^ ^16^ ^]^ Another study operating a bimetallic Mn‐Co catalyst with a 0.4 mg_PtRu_ cm^−2^ anode delivered a *P*_max_ of 1.1 W cm^−2^, but showed no stability performance in the manuscript.^[^ ^17^ ^]^

The development of single‐atom catalysts (SACs)—carbon‐supported metal nanoparticles where the active metals are exposed at the atomic level for enhanced interaction with the reactants, has been showing renewed promise in electrocatalysis.^[^ ^18^, ^19^, ^20^, ^21^, ^22^ ^]^ The independent active sites of SACs allow for distinct reaction pathways that differ from traditional heterogeneous catalysts due to their well‐defined coordination structures.^[^ ^23^, ^24^ ^]^ Moreover, the electron transfer between the substrate and metal centers could be enhanced owing to the strong metal‐support interactions.^[^ ^25^, ^26^ ^]^ In this context, PGM‐free SACs for the ORR in alkaline, particularly those containing Fe, have been studied, and they showed promising performance as AEMFC cathodes when combined with high‐loading PGM anodes (0.4–1 mg_PGM_ cm^−2^). However, when the anode PGM loading is reduced or eliminated, the cell performance suffers, therefore, reports in the literature are rare.^[^ ^27^, ^28^ ^]^

State‐of‐the‐art single‐atom PGM‐free ORR catalysts as commonly shown in the literature are synthesized using template materials such as ZIF‐8, a metal‐organic framework made from Zn and 2‐methylimidazoe.^[^ ^19^, ^29^, ^30^, ^31^ ^]^ The template acts as i) a chemical buffer to increase the concentration of FeN_4_ sites, and ii) a morphological stencil to control the metal particle size and prevent the formation of clustered Fe species.^[^ ^32^ ^]^ However, Zn‐based template synthesis methods require the removal of the template, which results in a considerable amount of inorganic byproduct and additional processing steps that increase synthesis complexity and cost. Furthermore, the volatilization of Zn during pyrolysis can lead to inconsistencies in active site distribution and may limit the scalability of the process. Additionally, most PGM‐free SACs used for ORR in alkaline media were shown in the literature to have enhanced electrocatalytic performance but could not meet the long‐term durability requirements for practical applications.^[^ ^33^ ^]^ These challenges highlight the need for alternative catalyst synthesis strategies that do not rely on template materials.^[^ ^19^, ^24^ ^]^ It is therefore extremely desirable to design cost‐effective SACs from earth‐abundant raw materials for ORR in alkaline media with superior performance and durability when integrated into advanced energy applications and devices.

In light of these challenges, we propose a CRM‐free SAC based on Fe additives and pyrolyzed polyimide (PI) nanoparticles followed by magnetic purification for ORR in alkaline environments. This approach circumvents the issues associated with template removal, reduces synthesis complexity, and enhances control over the final catalyst structure. The purified catalyst with single‐atom Fe centers shows enhanced ability to catalyze the ORR in H_2_|O_2_ AEMFCs as compared to the clustered, non‐purified catalyst (*P*_max_ ∼1.8 compared to 1.5 W cm^−2^ @ 80 °C). As cell stability reports at current densities higher than 600 mA cm^−2^ remain virtually non‐existent,^[^ ^10^, ^34^ ^]^ we tested this new system under a constant high current density load of 1000 mA cm^−2^ and it showed promising short‐term stability, with a slight decay of only 2 mV h^−1^ over 50 h. To the best of our knowledge, this is the first instance of a CRM‐free cathode catalyst demonstrating such a durability achievement at extremely high current densities in AEMFCs. With further architectural optimization of the anode loading down to 0.1 mg cm^−2^, we obtained anode PGM‐loading normalized specific power values as high as 11.3 W ${\mathrm{mg}}_{\mathrm{PGM}}^{-1}$ and current density values at 0.65 V exceeding 1.5 A cm^−2^. Lastly, we incorporate a CRM‐free NiMo/KB anode and operate at cell temperatures of 80 °C and higher, to investigate temperature‐induced reaction kinetics.^[^ ^35^, ^36^, ^37^, ^38^ ^]^ The latter results in the first fully CRM‐free high‐temperature (HT)‐AEMFC that operates at 118 °C and performs well with both O_2_ and ambient air, highlighting the versatility of this single‐atom CRM‐free cathode catalyst.

## 2 Morphological and Structural Characterization

**Figure 1:**
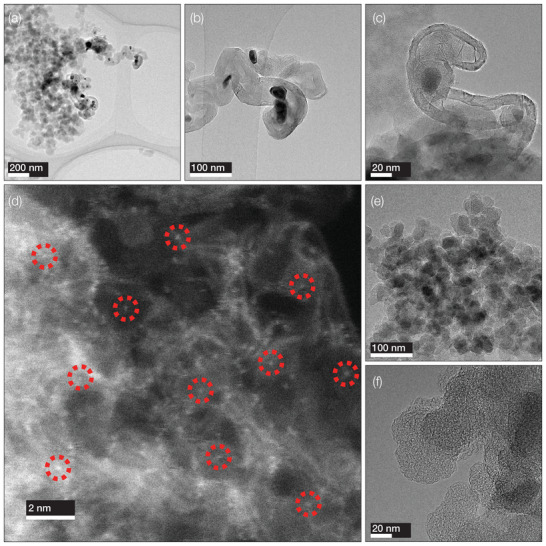
Microscopy images. a–c) TEM images of non‐purified Fe(SA + Nano)/PI catalyst, d) HAADF‐STEM image of the magnetically purified Fe(SA)/PI catalysts with single atoms identified by red‐circles and e,f) TEM images of the magnetically purified Fe(SA)/PI catalyst. Fe presence was proven by EDX signal shown in Figure S2a (Supporting Information).

To avoid the use of a chemical template to form single‐atom Fe centers,^[^ ^39^ ^]^ a magnetic purification technique was introduced to separate larger iron clusters.^[^ ^40^ ^]^ **Figure** **1** shows transmission electron microscopy (TEM) images of the polyimide nanoparticles after pyrolysis. The diameters of the polyimide particles post‐carbonization appear to be 50–100 nm, which is of a suitable size range for fuel cell applications. In Figure 1a–c the images of the non‐purified catalyst (Fe(SA + Nano)/PI) exhibit dark and faceted particles, which are Fe clusters formed by the reduction of the Fe precursors during the heat treatment. Scanning transmission electron microscopy (STEM) images confirm the cluster formations consist of a mixture of nanoparticles and single atoms (Figure S1a, Supporting Information). However, no Fe clusters were observed in the images for the purified catalyst (Fe(SA)/PI) in Figure 1e,f and Figure S1b (Supporting Information).

Atomic‐level high‐angle annular dark‐field (HAADF)‐STEM imaging and energy‐dispersive X‐ray (EDX) elemental maps further verify the presence of well‐dispersed single atoms distributed throughout the catalyst layer as shown in Figure 1d and Figures S1c,d (Supporting Information), respectively. The EDX Elemental spectra shown in Figure S2 (Supporting Information) display distinct Fe signals, namely Fe‐Kα, Fe‐Kβ, and Fe‐L, indicating the formation of highly dispersed Fe centers.

The Brunauer‐Emmett‐Teller (BET) surface areas were determined to be 1330 and 1416 m^2^ g^−1^ (**Table** **1**) for the samples before and after magnetic purification, respectively. Such high surface areas suggest the formation of micropores in the carbon support nanoparticles, which are derived from the etching effect of the NH_3_ treatment during the pyrolysis protocol.^[^ ^32^ ^]^ The chemical composition and nature of Fe species of both samples were determined by carbon, hydrogen, and nitrogen (CHN) elemental analysis and electron probe micro analysis (EPMA), and the results are summarized in Table 1. The data shows that the CHN contents before and after the magnetic purification were quite similar, but the Fe content in Fe(SA)/PI at 0.43% was noticeably lower than that of Fe(SA + Nano)/PI, at 0.67%, suggesting that some Fe species were eliminated. The Raman spectra of these two catalysts are shown in Figure S3 (Supporting Information). Distinct peaks at 1360 cm^−1^ (D‐band) and 1608 cm^−1^ (G‐band) were observed, with both catalysts exhibiting a similar intensity ratio (I_D_/I_G_ = 1). This indicates that their intrinsic electrical conductivity is at a comparable level.

**Table 1:** Elemental composition and specific surface areas of the Fe(SA + Nano)/PI and Fe(SA)/PI catalysts.

Sample name	Elemental analysis [wt.%]			Electron probe micro analysis [wt.%]	Specific surface area [m^2^/g]
	C	H	N	Fe	*A*_BET_
Fe(SA + Nano)/PI	93.1	0.3	2.7	0.67	1330
Fe(SA)/PI	93.1	0.4	2.7	0.43	1416

## 3 Materials Compositional Analysis

**Figure 2:**
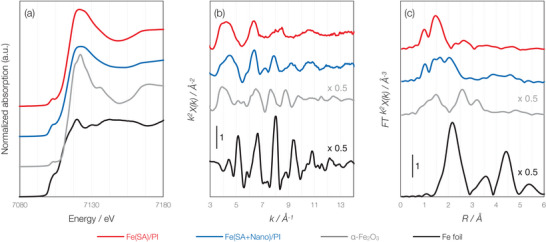
Fe K‐edge spectra. a) XANES, and *k^2^*‐weighted spectra showing b) EXAFS and c) FT‐XAFS of Fe(SA + Nano)/PI and Fe(SA)/PI, together with α‐Fe_2_O_3_ and Fe foil as references.

X‐ray absorption fine structure (XAFS) spectroscopy was carried out for further detailed analysis of the Fe species before and after the magnetic purification procedure. **Figure** **2**a shows the Fe K‐edge XANES spectra of Fe(SA + Nano)/PI and Fe(SA)/PI together with those of α‐Fe_2_O_3_ and Fe foil as references. The energy at 0.5 of normalized absorption, which is referred to as the edge energy, was 7111.4 eV for Fe(Nano + SA)/PI and 7112.3 eV for Fe(SA)/PI. The edge energy of Fe(SA)/PI was similar to that of α‐Fe_2_O_3_ (7112.7 eV) but slightly larger than Fe(SA + Nano)/PI, which suggests that Fe metal species in Fe(SA)/PI were removed by the purification procedure and that most of Fe species are in Fe^3 +^ state.

The *k* ^2^‐weighted extended XAFS (EXAFS) spectra of Fe(SA + Nano)/PI and Fe(SA)/PI are shown in Figure 2b. The oscillation pattern of Fe(SA + Nano)/PI at > 9 Å^−1^ resembles that of Fe foil, suggesting the formation of Fe metal particles. In contrast, the EXAFS spectrum of Fe(SA)/PI does not exhibit such oscillations.

Figure 2c displays the Fourier‐transforms of the EXAFS spectra (FT‐EXAFS). The FT‐EXAFS spectrum of Fe(SA)/PI exhibits a peak at 1.5 Å, which is assignable to Fe‐N scattering. In fact, the spectrum is well‐fitted with a FeN_4_ structure (Table S1, Supporting Information). The FT‐EXAFS spectrum of Fe(Nano + SA)/PI presented a peak at 2.0 Å due to Fe‐Fe scattering of Fe metal in addition to the peak at 1.5 Å due to Fe‐N scattering. These results confirm that almost all of the Fe species in Fe(SA)/PI exist as Fe single atoms having a FeN_4_ structure, whereas Fe(SA + Nano)/PI contains a mixture of Fe metal nanoparticles and Fe single atoms.

## 4 Electrochemical Analysis

**Figure 3:**
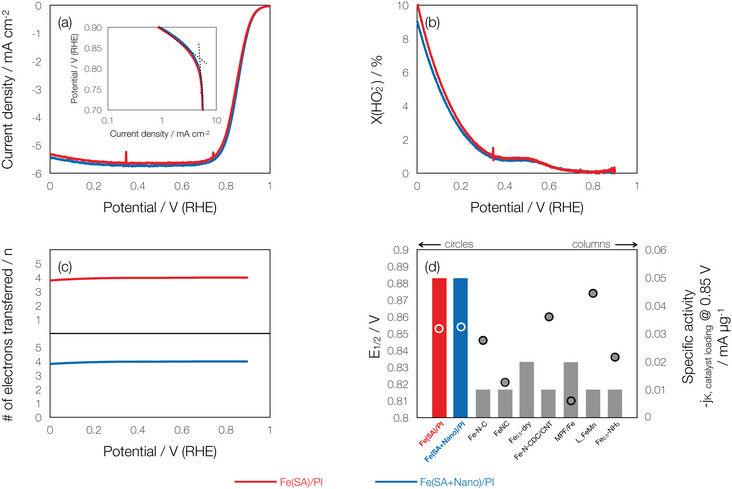
RRDE voltammograms of Fe(SA + Nano)/PI and Fe(SA)/PI. a) ORR polarization curves with Tafel plots (Inset), b) % peroxide generation, c) # of electrons transferred, and d) half‐wave potential (circles) and specific activity (kinetic current density at 0.85 V normalized to the total catalyst loading used during RRDE measurement, columns). All measurements were performed in 0.1 m KOH aqueous electrolyte at 1600 rpm with catalyst loadings of 60 µg cm^−2^.

The electrocatalytic activity of the catalysts before and after the purification was evaluated by rotating ring‐disk electrode (RRDE) voltammetry in a 0.1 m KOH solution. **Figure** **3**a–c shows the oxygen reduction current, peroxide selectivity and number of electrons transferred, respectively. Both measured catalyst samples showed similar activity for all parameters. It should be noted that the peroxide selectivity values of ∼0.45% at 0.6 V are extremely low, considering the higher values in recent reports within the past 5 years (**Table** **2**). A closer look at the high potential regions (Tafel plots shown in the inset to Figure 3a) indicates similar kinetic activity, which suggests that both catalysts would exhibit similar performance as ORR electrocatalysts in AEMFCs.

**Table 2:** Catalytic performance of the Fe(SA)/PI and Fe(SA + Nano)/PI catalysts in alkaline media in comparison to the state‐of‐the‐art single atom Fe‐based PGM‐free cathode catalysts.

Catalyst	Loading [µg cm^−2^]	E_0_ [V] ^a)^	E_1/2_ [V]	− j_k,catalyst loading_ @ 0.85 V [mA µg^−1^]	X at 0.6 V	Refs.
Fe(SA)/PI	60	0.958	0.853	0.05	0.43%	This study
Fe(SA + Nano)/PI	60	0.959	0.854	0.05	0.44%	This study
Fe‐N‐C	200	0.960	0.846	0.01	< 1%	[15]
Fe‐N‐C	–	0.970	0.89	–	2.19%	[30]
FeNC	100	0.931	0.821	0.01	–	[46]
Fe_0.5_‐dry	200	0.990	–	0.02	1.15%	[29]
Fe‐N‐CDC/CNT	400	0.990	0.860	0.01	6.10%	[47]
hp‐FeNC‐20	–	0.980	0.889	–	–	[31]
MPF/Fe	80	0.940	0.810	0.02	–	[48]
L_FeMn	600	0.942	0.874	0.01	1.95%	[49]
Ag_NPs_@Fe‐N‐C	–	1.063	0.885	–	2.56	[50]
Fe_0.5_‐NH_3_	200	0.978	0.836	0.01	4.47	[51]

^a)^ The potential for 0.1 mA cm^−2^ of current density.

The catalytic activity in the present study is compared to those of other state‐of‐the‐art Fe‐based catalysts in the literature (Table 2). The onset potentials (E_0_) in this study were ∼0.959 V at a 60 µg cm^−2^ catalyst loading, whereas similar E_0_ values have been reported with higher catalyst loadings. The half‐wave potentials (E_1/2_) of both catalysts are fairly similar at 0.853 and 0.854 V for the Fe(SA)/PI and Fe(SA + Nano)/PI, respectively, and more positive than other Fe‐based SACs (Figure 3d). Moreover, Figure 3d depicts that the specific activities of the catalysts measured at 0.85 V were as much as 5× that of other catalysts suggesting that the catalytic activity of Fe(SA)/PI and Fe(SA + Nano)/PI for ORR is quite high and well‐suited for the 4‐electron ORR pathway as corroborated by Figure 3c.

Given that the electrochemical activity of the catalysts was very similar, we employed density functional theory (DFT) to gain deeper insights into the ORR reaction mechanism. Based on XAFS results, Fe(SA)/PI catalyst was chosen as a model catalyst with a single Fe atom anchored on nitrogen‐doped carbon with a FeN_4_ structure (Figure S4a, Supporting Information). As shown in Figure S4b (Supporting Information), the charge density of FeN_4_ is symmetrically distributed. Due to the difference in electronegativity, electrons are transferred from the central Fe atom to the surrounding N atoms, as illustrated in the charge density difference map in Figure S5 (Supporting Information). According to Bader charge analysis, the Fe atom transfers 0.9647 electrons to the surrounding N atoms, which tunes the electronic structure of the central Fe atom. Subsequently, the ORR reaction pathway on FeN_4_ was explored. In alkaline media, ORR involves the following elementary steps,^[^ ^41^, ^42^ ^]^ where the symbol “*” refers to the active site and (g) and (l) stand for gas and liquid phases, respectively.

(1) $$O_{2}\left(g\right)+H_{2}O\left(l\right)+e^{-}+\;*\;\to \mathrm{OO}H^{*}+OH^{-}$$

(2) $$\mathrm{OO}H^{*}+e^{-}\to O^{*}+OH^{-}$$

(3) $$O^{*}+H_{2}O\left(l\right)+e^{-}\to OH^{*}+OH^{-}$$

(4) $$OH^{*}+e^{-}\to \;*+\;OH^{-}$$

The Gibbs free energy for each elementary step of ORR on Fe(SA)/PI was calculated. The optimized structure of the ORR intermediates (OOH*, O*, and OH*) on Fe(SA)/PI is shown in Figure S6 (Supporting Information). As can be seen, all electron‐transfer steps on Fe(SA)/PI are exothermic at U = 0 V. The Gibbs free energy change (ΔG) for the final step of OH* reduction to OH^−^ is the smallest among the four elementary reaction steps and is considered the rate‐determining step for ORR, which is consistent with the computational results for Fe single‐atom catalysts in previous reports.^[^ ^43^, ^44^, ^45^ ^]^

## 5 Catalysts’ Performance in Membrane Electrode Assemblies (MEAs)

MEAs were constructed with radiation‐grafted ethylene tetrafluoroethylene (ETFE) AEMs,^[^ ^52^, ^53^, ^54^, ^55^ ^]^ PtRu/C based anodes and the Fe(SA + Nano)/PI and Fe(SA)/PI catalysts used as cathodes according to the procedures reported elsewhere.^[^ ^36^, ^56^, ^57^ ^]^ Before directly comparing the performance of the Fe(SA + Nano)/PI and Fe(SA)/PI cathodes, the purified Fe(SA)/PI was tested at different loadings to determine the optimal catalyst loading, while keeping the anode PGM catalyst and loading constant at 0.6 mg_PtRu_ cm^−2^. At 60 °C without back‐pressurization under H_2_|O_2_ AEMFCs, the best cathode loading was found to be 1.5 mg_Total catalyst_ cm^−2^ (Figure S7, Supporting Information). Although similar peak power and limiting current densities were obtained for the 1.0 and 1.5 mg_Total catalyst_ cm^−2^ loading cells, a closer look at the kinetic region shows that the 1.5 mg_Total catalyst_ cm^−2^ based cell had a slightly improved open circuit voltage (OCV) and enhanced current densities at all voltages (Figure S7c, Supporting Information). This may be attributed to better access to the active sites owing to the slightly higher loading. When the loading was increased to 3 mg_Total catalyst_ cm^−2^, the ohmic and mass transport region performances decreased indicative of possible constituent transport and flooding issues.

The polarization curves and power density curves of the AEMFCs using Fe(SA)/PI and Fe(SA + Nano)/PI cathodes with anodes having PGM loadings of 0.6 mg_PtRu_ cm^−2^ are shown in Figure S8a (Supporting Information). When operated at 60 °C without back‐pressurization under H_2_|O_2_, the OCVs of both cells are above 0.9 V, with a favorable shift of 70 mV for the cell with the Fe(SA)/PI cathode (Figure S8b, Supporting Information). The improved OCV translated to comparatively higher kinetic and ohmic region performances as well. In addition, the *P*_max_ of the cell containing Fe(SA)/PI in the cathode arrived at 1.0 W cm^−2^ which was ∼30% higher than the non‐purified catalyst cell. These differences in *P*_max_ can be attributed to the lower BET surface area of the Fe(SA + Nano)/PI (Table 1), which could account for potential active site and pore blockage due to clustering caused by the lack of purification.

**Figure 4:**
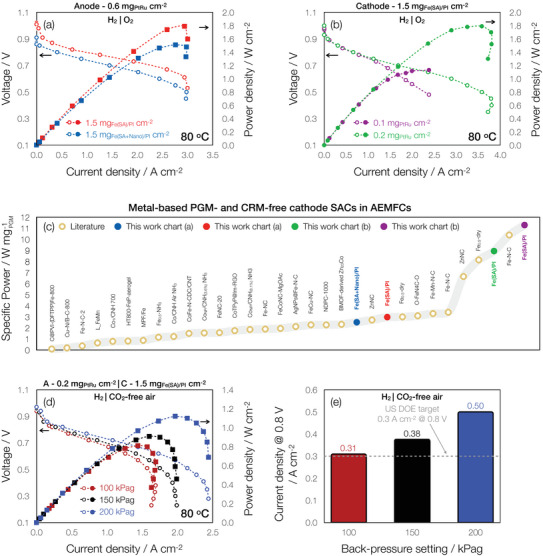
AEMFC polarization performance. H_2_|O_2_ polarization curves (V, empty symbols, Y1 axis) and power density curves (filled symbols, Y2 axis) as a function of current density for AEMFCs tested at 80 °C with 200 kPag of back‐pressurization and dewpoints set at 74 and 76  C on the anode and cathode, respectively with a) Fe(SA)/PI and Fe(SA + Nano)/PI cathodes with PtRu/C anode loadings of 0.6 mg_PtRu_ cm^−2^ and b) Fe(SA)/PI cathodes with PtRu/C anode loadings of 0.2 and 0.1 mg_PtRu_ cm^−2^. c) Performance comparison with literature‐based^[^ ^15^, ^16^, ^29^, ^30^, ^31^, ^46^, ^47^, ^48^, ^49^, ^50^, ^51^, ^57^, ^67^, ^68^, ^69^, ^70^, ^71^, ^72^, ^73^, ^74^, ^75^, ^76^ ^]^ single atom metal‐based PGM‐ and CRM‐free cathode catalysts in AEMFCs within the past 5 years containing PGM‐based anodes and operated with H_2_|O_2_. d) H_2_|CO_2_‐free air polarization (V, empty symbols, Y1 axis) and power density (filled symbols, Y2 axis) curves as a function of current density at 80 °C with full humidification and 100, 150, and 200 kPag of back‐pressurization on both anode and cathode, respectively and e) comparison of current densities at 0.8 V. The dashed gray line denotes the US DOE target for 2025.^[^ ^34^ ^]^ For (d) and (e), the anode loadings were 0.2 mg_PtRu_ cm^−2^. For all AEMFCs, the cathode loadings were 1.5 mg_Total catalyst_ cm^−2^.

The results of increasing the cell temperature to 80 °C and adding back‐pressure under H_2_|O_2_ can be seen in **Figure** **4**a. At the increased temperature, similar high OCV, kinetic, and ohmic region performances were realized for the Fe(SA)/PI cathode cell compared to the Fe(SA + Nano)/PI cathode cell, despite the performances between the catalysts being indistinguishable at the RRDE level. We hypothesize that the differences in OCV between RRDE and AEMFC tests are due to the difference in ionomers used at each step. To accurately compare to the literature at the RRDE level, we used Nafion as the ionomer, a common method to evaluate ORR catalysts in alkaline media,^[^ ^58^, ^59^ ^]^ whereas for the AEMFC tests we used Fumion anion exchange ionomer (AEI). According to earlier research, AEIs containing quaternary ammonium cationic groups can induce the formation of an ionomeric layer on the surface of the catalytically active particles, which acts as a barrier that affects the catalyst layer porosity and gas permeability.^[^ ^60^, ^61^, ^62^ ^]^ This phenomenon may have made the less defined active sites of the Fe(SA + Nano)/PI cathode cell difficult to reach, which had a discernible effect on the OCV since reduced cathode kinetics increased the cell's vulnerability to hydrogen crossover and excessive cathode polarization.^[^ ^63^, ^64^ ^]^

Along with a higher OCV value, the Fe(SA)/PI cathode cell delivers better performance than the Fe(SA + Nano)/PI cathode cell in both the kinetic and ohmic regions of the i‐V curve (Figure 4a), despite exhibiting similar performances in RRDE. This can be attributed to the inherent differences between the RRDE liquid electrolyte and FC test environments.^[^ ^65^ ^]^ Specifically, the RRDE tests in our work were conducted at room temperature in 0.1 m KOH aqueous electrolyte, while the AEMFC tests were conducted at 80 °C with O_2_ and H_2_ humidified gases at 76 and 74 °C, respectively, and back‐pressurized at 200 kPag in both electrodes. As a result, the Fe(SA)/PI cell outperformed the Fe(SA + Nano)/PI cell in terms of *P*_max_ (1.8 compared to 1.5 W cm^−2^) and both cells arrived at limiting current density values as high as 3 A cm^−2^. At 0.8 V, the measured current density of the Fe(SA)/PI AEMFC was close to 1 A cm^−2^ and arrived at almost 3 A cm^−2^ when measured at 0.65 V, which, to the best of our knowledge, is the highest in the literature in comparison to other single‐atom Fe‐based cathode catalysts in AEMFCs (Table S2, Supporting Information). By comparison, a state‐of‐the‐art single atom Fe‐N‐C catalyst from Pajarito Powder^[^ ^15^ ^]^ with a similar PtRu anode loading of 0.6 mg_PtRu_ cm^−2^ exhibited a current density of 0.57 A cm^−2^ @ 0.8 V and 2.1 A cm^−2^ @ 0.65 V while the current density of our Fe(SA)/PI cell measured up to 60% greater at the same voltages.

Figure 4b shows the polarization and power density curves of the cells with reduced PGM anode loadings (0.2 and 0.1 mg_PtRu_ cm^−2^) and cathodes loaded to 1.5 mg_Fe(SA)/PI_ cm^−2^, at the same test conditions as Figure 4a. Remarkably, the AEMFC with the 0.2 mg_PtRu_ cm^−2^ arrived at the same high *P*_max_ (1.8 W cm^−2^) and a higher limiting current density above 3.5 A cm^−2^ than the Fe(SA)/PI AEMFC with the higher anode loading in Figure 4a. Furthermore, the AEMFC with 0.2 mg_PtRu_ cm^−2^ arrived at a current density of 2.36 A cm^−2^, which was almost 2.5× greater than the 2022 US DOE target of 1 A cm^−2^ @ 0.65 V,^[^ ^66^ ^]^ and exceeded other recent reports in the literature (Table S2, Supporting Information).

When the anode PGM loading was further lowered to 0.1 mg_PtRu_ cm^−2^ i.e. within the US DOE PGM loading target,^[^ ^66^ ^]^ the *P*_max_ dropped by ∼37%, down to 1.13 W cm^−2^. Nevertheless, the calculated specific power based on the remaining PGM in the anode was 11.3 W ${\mathrm{mg}}_{\mathrm{PGM}}^{-1}$, the highest in the literature among metal‐based PGM‐free and to a greater extent CRM‐free cathode SACs in AEMFCs, as shown in Figure 4c and Table S2 (Supporting Information).

The cathode kinetics of the ORR in alkaline environments of AEMFCs are significantly more favorable, leading to competitive performances of PGM‐free catalysts akin to their PGM‐based counterparts. However, their loadings are usually higher owing to their decreased activities relative to PGMs, indicating that evaluating their intrinsic activities within the cell environment is a crucial step in determining the efficacy of catalyst design. Figure S9 (Supporting Information) depicts the kinetic activity of Fe(SA)/PI‐based cathode cells with low‐loading anodes from Figure 4b at full humidification. At 0.9 V_iR − free_, the AEMFCs generated current densities of 104 (0.2 mg_PtRu_ cm^−2^) and 70 mA cm^−2^ (0.1 mg_PtRu_ cm^−2^) respectively, significantly above the 2025 target of 44.0 mA cm^−2^ according to the recommended testing protocol of the US DOE.^[^ ^34^ ^]^ When the oxidant was switched to CO_2_‐free air, *P*_max_ values as high as 1.2 W cm^−2^ (Figure 4d) were obtained and current densities measuring up to 0.5 A cm^−2^ at 0.8 V surpassing the US DOE target of 0.3 A cm^−2^ (Figure 4e).^[^ ^34^ ^]^

## 6 Durability Performance as CRM‐Free Cathodes in AEMFCs

One of the requirements for fuel cells is that they exhibit stable and reliable performance over time. Given that cell components influence the initial and long‐term performance it is critical to evaluate both metrics in comparison to the literature. In terms of recent studies within the past five years of metal‐based PGM‐ and CRM‐free cathode SACs for AEMFCs, only a few studies show durability data as shown in Figure 6a,b and Table S2 (Supporting Information). Three of these studies were operated at a constant current density of 600 A cm^−2^,^[^ ^15^, ^46^, ^47^ ^]^ showing promising durability with overall voltage degradations of 10% or less. Reports operated at a slightly lower current density of 400 A cm^−2^,^[^ ^30^, ^73^, ^74^ ^]^ exhibited overall voltage degradations as high as 63%. The remaining reports were evaluated at 0.25 and 200 A cm^−2^, resulting in overall degradation rates of 25%^[^ ^16^ ^]^ and 21%,^[^ ^75^ ^]^ respectively. These varied results provide insight into the challenges experienced when transitioning from PGM‐based to PGM‐ and CRM‐free catalyst materials.

**Figure 5:**
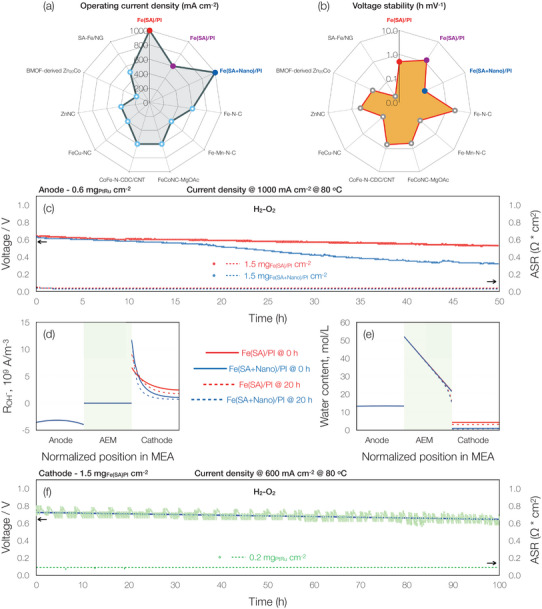
AEMFC durability performance tests. a,b) Radar charts of our work compared to single atom metal‐based PGM‐ and CRM‐free cathode catalysts in AEMFCs with PGM‐based anodes operated with H_2_|O_2_ in the past 5 years.^[^ ^15^, ^16^, ^30^, ^46^, ^47^, ^73^, ^74^, ^75^ ^]^ Chart (a) shows the constant current densities used for the durability tests and (b) stability over time depicting how long before cell performance would decrease by 1 mV; higher values indicate better stability, c) 50‐h durability tests of AEMFCs from Figure 4a at a constant current density of 1000 mA cm^−2^, simulated d) reaction distributions and e) water content profiles for durability tests in (c) at different operating times and f) 100‐h durability test of Fe(SA)/PI cathode at a constant current density of 600 mA cm^−2^ with 0.2 mg_PtRu_ cm^−2^ in the anode. For (c&f), the cathode loadings were 1.5 mg_Total catalyst_ cm^−2^, and test conditions were 80 °C with 150 kPag of back‐pressurization and dewpoints set at 74 and 76 °C on the anode and cathode, respectively.

Given that our Fe(SA)/PI AEMFCs with 0.6 and 0.2 mg_PtRu_ cm^−2^ anode loadings exhibited high initial performances, we conducted some durability experiments on the cells directly after obtaining the polarization curves. At a current density hold of 1000 mA cm^−2^, the AEMFC with the Fe(SA)/PI cathode exhibited an overall voltage degradation rate of 2 mV h^−1^ (18% overall reduction), compared to the 7 mV h^−1^ (54% overall reduction) of the Fe(SA + Nano)/PI cathode AEMFC after 50‐h (**Figure** **5**c). Considering that both cells were assembled and tested similarly, with the only difference being the cathode catalyst, we propose that the reduced durability of the Fe(SA + Nano)/PI cathode cell was due to impaired ORR kinetics which enhanced chemical degradation of the ionomer in the cathode catalyst layer (CCL) over time.^[^ ^10^, ^77^ ^]^ A closer examination of the ASR values in Figure S10 (Supporting Information) for both cells revealed almost no increases over the brief 50 h period, suggesting that the ionic conductivity was adequately maintained throughout the experiments. Therefore, we employed a 1D numerical model^[^ ^78^, ^79^ ^]^ to simulate the cells’ performance by concentrating on the hydration and reaction profiles across the cells.

The findings showed that the reaction distribution profiles (Figure 5d) and hydration levels (Figure 5e) in the anode and AEM were comparable for both cells at the start of the tests (i.e., T = 0 h). As for the cathodes, the ORR occurred mostly near the AEM‐CCL interface of the Fe(SA + Nano)/PI cathode cell, represented by the parabolic shape (solid blue line, Figure 5d). This is in contrast to the Fe(SA)/PI cathode cell (solid red line with the milder slope in Figure 5d) indicating that the ORR was more uniformly distributed along the entire CCL. Consequentially, the Fe(SA + Nano)/PI cathode cell consumed more water close to the AEM‐CCL interface, which led to a lower degree of hydration of the rest of the CCL.

We examined the hydration levels and reaction distribution profiles once again at T = 20 h, following the onset of the decline in long‐term stability performance of the Fe(SA + Nano)/PI cathode cell. The findings revealed no changes in the reaction distribution profiles (Figure 5d S5d) and hydration levels (Figure 5e) of the anodes. In the case of the AEMs, the reaction distribution profiles (Figure 5d) also showed no changes, supporting the theory behind the ASR values remaining constant (Figure S10, Supporting Information). However, reductions in the reaction distribution profiles in the cathodes of both cells can be seen, indicating a change in the ORR kinetics (Figure 5d). The dashed red line of the Fe(SA)/PI cathode cell still demonstrated a more uniform distribution of the reactions across the breadth of the catalyst layer, but the dashed blue line of the Fe(SA + Nano)/PI cathode cell maintained a parabolic shape, indicating that higher levels of water consumption continued at the AEM‐CCL interface.

Variations in hydration and reaction distribution profiles can negatively impact a cell during operation due to: i) increased kinetic overpotential i.e when inhomogeneous reaction distributions cause localized degradation of the ionomer owing to insufficient hydration and ii) poor catalyst utilization whereby the ORR is restricted to the AEM‐CCL interface rather than being distributed throughout the catalyst layer. We believe that the combination of these effects accelerated the chemical degradation of the ionomer near the membrane and significantly impaired the Fe(SA + Nano)/PI cathode cell's operation. In contrast, the Fe(SA)/PI cathode cell exhibited a more uniform reaction distribution across the cell over time, enabling full catalyst utilization in the CCL. This resulted in more balanced water consumption, preventing excessive degradation near the AEM‐CCL interface leading to better overall hydration and higher stability.

**Figure 6:**
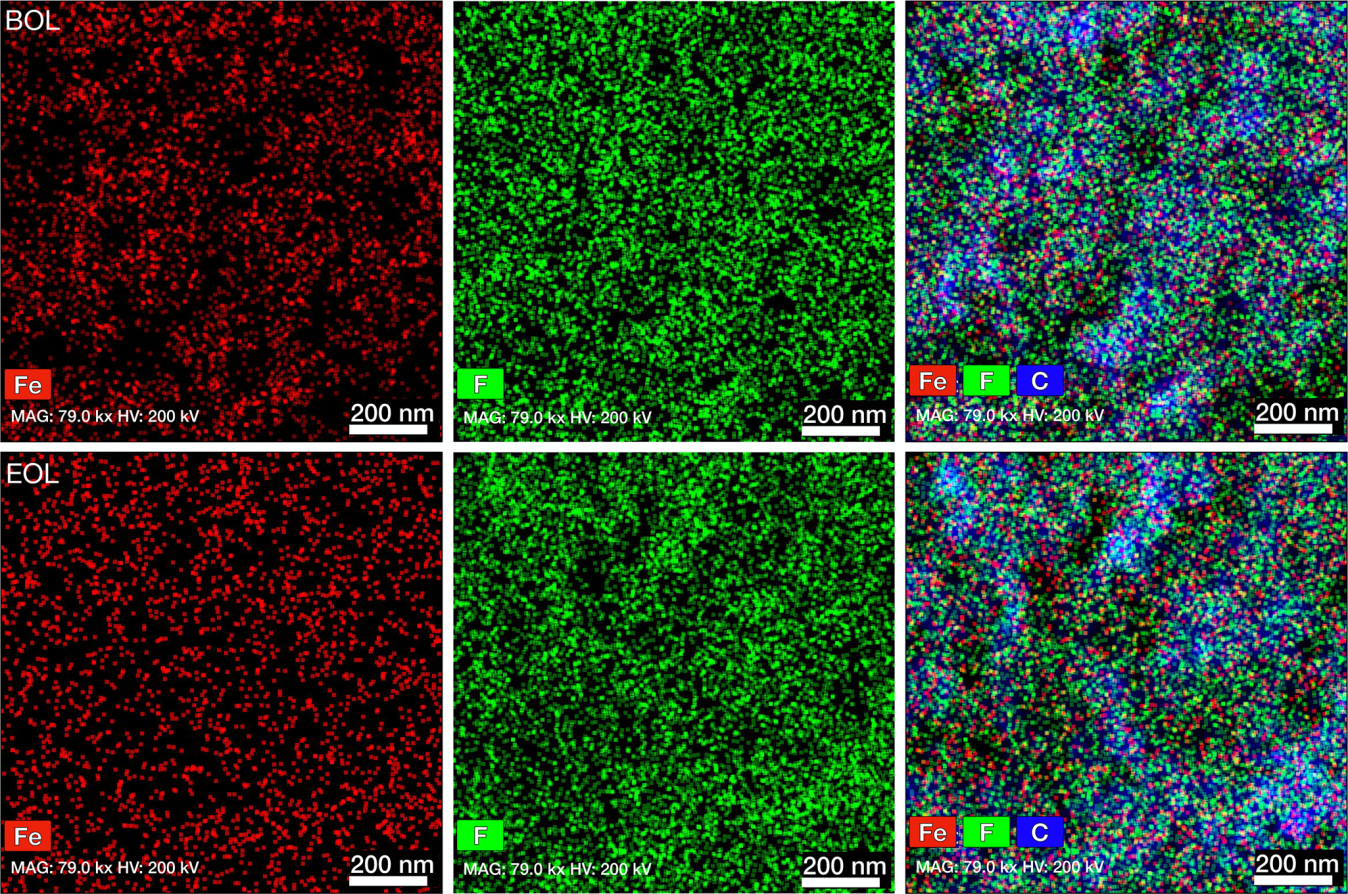
Catalyst layer images. STEM‐EDX images of a Fe(SA)/PI catalyst layers, both before and after durability testing at 1000 mA cm^−2^ from Figure 5c, showing Fe, F and Fe/F/C elemental maps. The top row of images shows the beginning of life (BOL) and the bottom row shows the end of life (EOL).

As our literature study in Table S2 (Supporting Information) demonstrates, operating PGM‐free cathode AEMFCs at such a high current density of 1000 mA cm^−2^ is uncommon. By comparison to Fe(SA)/PI cathode cell's 2 mV h^−1^ after 50 h performance, a recent and excellent report with PGM‐based catalysts in both electrodes evaluated at 1000 mA cm^−2^ for 24 h, experienced an overall voltage reduction of 4.2 mV h^−1^.^[^ ^80^ ^]^ Therefore, we used HAADF STEM‐EDX to further examine the fidelity of the Fe(SA)/PI CCL both prior to and after durability testing. Beginning‐of‐life (BOL) images in the top row of **Figure** **6** show that both iron and fluorine particles are well dispersed over the carbon support. After the 50 h test, the end‐of‐life (EOL) images (bottom row of Figure 6), show a slight loss of Fe and F particles, which can be attributed to the chemical degradation of the ionomer that led to the eventual loss of active sites. This is confirmed by the before and after EDX spectra (Figure S11, Supporting Information), which indicates a decrease in Fe content. These findings verify that, in comparison to the non‐purified catalyst, the ORR process was much enhanced by the well‐defined single‐atom catalytic sites of the purified Fe(SA)/PI. We suggest that employing common voltage recovery techniques such as polarization curves to refresh the water in the cell as well as optimizing the gas dew point temperatures and/or gas flow rates could potentially improve the cell durability further,^[^ ^10^ ^]^ but these techniques were out of scope for this study as the objective was to test the performance of the catalysts without adding any biases.

Promising stability is also seen with the reduced anode PGM loading and lower current density of 600 mA cm^−2^. As seen in Figure 5f during a 100‐h test, the Fe(SA)/PI cathode cell experienced an overall voltage degradation rate of 0.8 mV h^−1^ (7% reduction from the original value), one of the lowest values among single‐atom PGM‐free Fe‐based cathode AEMFCs in the literature (Table S2, Supporting Information).

## 7 Performance in a CRM‐Free High‐Temperature (HT)‐AEMFC

**Figure 7:**
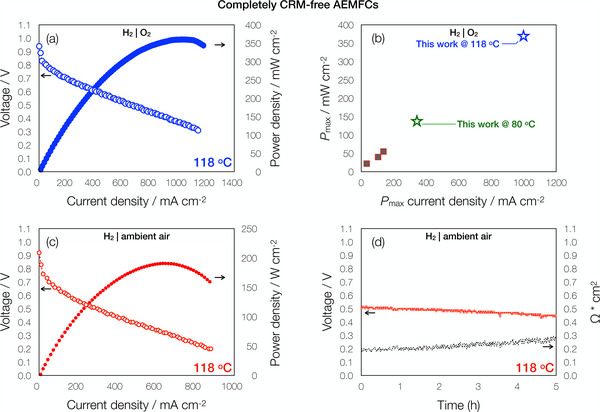
Performance of completely CRM‐free HT‐AEMFCs. a) H_2_|O_2_ polarization curves (V, empty symbols, Y1 axis) and power density curves (filled symbols, Y2 axis) as a function of the current density of completely CRM‐free HT‐AEMFC and b) performance comparison of our completely CRM‐free AEMFCs against other completely CRM‐free catalyst AEMFCs reported in the literature^[^ ^82^, ^83^, ^84^ ^]^ operated with H_2_|O_2_, comparing peak power density values and the current density at which it was obtained. H_2_|ambient air c) polarization curves (V, empty symbols, Y1 axis) and power density curves (filled symbols, Y2 axis) as a function of the current density of completely CRM‐free HT‐AEMFC and d) a 5‐h short‐term durability test. The HT‐AEMFC is made with a 1.5 mg_Fe(SA)/PI_ cm^−2^ cathode and NiMo/KB anode loaded to 13 mg_NiMo_ cm^−2^ and operated at 118 °C with 250 kPag of back‐pressurization and dewpoints of 116 and 117 °C on anode and cathode, respectively.

The ultimate objective of AEMFC technology is to function completely without the use of CRMs with ambient air as the oxidant. Therefore, we swapped the PtRu/C anodes in our cells for a CRM‐free NiMo/KB anode (loading of 13 mg_NiMo_ cm^−2^) to create a completely CRM‐free cell. This is an additional test of critical importance because the long‐term deployment of AEMFCs is hinged upon the reduction of CRMs (which includes PGMs)^[^ ^81^ ^]^ and currently, less than 0.1% of all studies reporting AEMFC performance are completely CRM‐free.^[^ ^82^, ^83^, ^84^ ^]^ Figure S12a (Supporting Information) shows the polarization and power density curves of the completely CRM‐free AEMFC measured at 80 °C, while **Figure** **7**a shows the HT‐AEMFC performance at 118 °C. As seen from the figures, the 38 °C rise in cell operating temperature yielded a significant 276% increase in *P*_max_ from 135 to 372 mW cm^−2^. Furthermore, the HT‐AEMFC arrives at the *P*_max_ at an impressive current density of ∼1000 mA cm^−2^, which is the highest among all PGM‐ and CRM‐free AEMFC reports in the literature operated with H_2_|O_2_ (Figure 7b, Figure S12b and Table S3, Supporting Information).

Among the CRM‐free AEMFC reports in the literature, there is only one account displaying performance with ambient air. In the work, the researchers arrive at a *P*_max_ of 27.5 mW cm^−2^ at a corresponding current density of 65 mA cm^−2^.^[^ ^83^ ^]^ Given that ambient air operation is imperative for AEMFC technology as successful integration without the addition of upstream carbon sequestration subsystems or infrastructure to facilitate CO_2_‐free air could lower the overall balance of plant cost, the burden of proof remains to be seen whether high‐temperature operation could subvert the deleterious effects of atmospheric CO_2_.^[^ ^85^, ^86^ ^]^ A switch of the cathode oxidant to ambient air in our completely CRM‐free HT‐AEMFC at 118 °C results in a *P*_max_ of 206 mW cm^−2^ at a current density of 652 mA cm^−2^ (Figure 7c) and promising stability with a slight reduction from 0.50 to 0.45 V after 5‐h at a constant current density of 225 mA cm^−2^ (Figure 7d). This is significant to note as the current density at which the *P*_max_ of our cell was achieved is 10× greater the single ambient air CRM‐free report in the literature (Table S4, Supporting Information) and lends credence to the potential of HT‐AEMFCs to abate the effects of CO_2._ All in all, this promising performance displays the versatility of the Fe(SA)/PI catalyst in AEMFCs and could apply to other electrochemical energy technologies that are necessary to maintain a clean and renewable environment.

## 8 Conclusion

A PGM‐free Fe‐based single‐atom catalyst was prepared by pyrolyzing polyimide nanoparticles with a small quantity of Fe additives in the absence of a sacrificial template. The catalyst was further purified using a magnetic field to remove Fe clusters, and the resulting morphology of the spherical catalyst particles was less than 100 nm in diameter. XAFS analysis confirmed the existence of Fe single atoms having FeN_4_ structure and RRDE tests in alkaline media revealed that the SAC exhibited high ORR activity and extremely low peroxide selectivity, indicating that it is well‐suited for the 4‐electron alkaline ORR pathway. Integration of the PGM‐free SAC into the cathode of a H_2_|O_2_ AEMFC yielded an impressive *P*_max_ of ∼1.8 W cm^−2^ @ 80 °C and promising short‐term durability, with a slight decay of only 2 mV h^−1^ over a 50 h period under a very high constant current density of 1000 mA cm^−2^. To the best of our knowledge, this is the first time that such a durability achievement has been shown in AEMFCs for a PGM‐free cathode catalyst. Further optimizations of the anode PGM loading resulted in PGM‐loading normalized specific power values as high as 11.3 W ${\mathrm{mg}}_{\mathrm{PGM}}^{-1}$. Finally, the potential of completely CRM‐free operation was shown in an HT‐AEMFC at 118 °C in O_2_ and ambient air with good performance and durability. The ambient air performance gives hope for future AEMFC studies at high temperatures to verify the reduction of the deleterious effects of CO_2_. These results show an effective approach to catalyst design to produce SACs with high activity and durability with promising prospects toward PGM‐ and CRM‐free integration into the next generation of high‐performing AEMFCs and HT‐AEMFCs.

## Conflict of Interest

The authors declare no conflict of interest.

## Author Contributions

Conceptualization by J.C.D., Y.N., and D.R.D.; data collection by J.C.D., H.N., S.N., S.W.‐C., J.Z., S.M.Z. and A.O.G., O.S., M.T.; data analysis by J.C.D., H.N., S.N., S.W.‐C., J.Z., S.M.Z., A.O.G. and C.W. O.S., M.T., K.Y.; writing—original draft by J.C.D., H.N., S.N. and Y.N.; writing—review, editing and approval of final version by J.C.D., H.N., S.N., S.W.‐C., J.Z., J.O., J.H., S.M.Z., A.O.G., C.W., O.S., M.T., K.Y., J.J., C.E.D., Y.N., and D.R.D.; funding acquisition by Y.N., and D.R.D.; supervision by Y.N., and D.R.D.

## Supporting information



Supporting Information

## Data Availability

The data that support the findings of this study are openly available in figshare.com at https://doi.org/10.6084/m9.figshare.28573574, reference number^[^ ^87^ ^]^.
