# Supplementary material for: Template‐Free Fabrication of Single Atom Fe‐Based Cathodes Unlock High‐Performing Anion‐Exchange Membrane Fuel Cells
Source: Adv Sci (Weinh). 2025 Jul 17;12(38):e01016. doi: 10.1002/advs.202501016 (PMC12520578; doi:10.1002/advs.202501016)
Supplement: Supplementary file 1 — Supporting Information [file ADVS-12-e01016-s001.pdf]

ADVANCED SCIENCE
Open Access

Supporting Information

for *Adv. Sci.*, DOI 10.1002/advs.202501016

Template-Free Fabrication of Single Atom Fe-Based Cathodes Unlock High-Performing Anion-Exchange Membrane Fuel Cells

John C. Douglin, Hideo Notsu, Shinsuke Nagata, Sapir Willdorf-Cohen, Jinliu Zhong, Junya Ohyama, Jiawei Hu, Syeda M. Zahan, Andres O. Godoy, Changlai Wang, Oluwafemi Sanumi, Masayuki Tsushida, Karam Yassin, Jasna Jankovic, Charles E. Diesendruck, Yuta Nabae and Dario R. Dekel**

Supporting Information

Template-Free Fabrication of Single Atom Fe-Based Cathodes Unlock High-Performing Anion-Exchange Membrane Fuel Cells

John C. Douglin, Hideo Notsu, Shinsuke Nagata, Sapir Willdorf-Cohen, Jinliu Zhong, Junya Ohyama, Jiawei Hu, Syeda M. Zahan, Andres O. Godoy, Changlai Wang, Oluwafemi Sanumi, Masayuki Tsushida, Karam Yassin, Jasna Jankovic, Charles E. Diesendruck, Yuta Nabae, and Dario R. Dekel**

Experimental Procedures

Chemicals and materials

Pyromellitic dianhydride (PMDA) was obtained from Tokyo Chemical Industry (TCI) and purified by sublimation. Hydrogen, Oxygen, CO_2-free air, ambient air, and Nitrogen gases with 99.999% purity were obtained from Maxima. Toray Carbon Paper 060 with 20% wet-proofing, used as gas diffusion layers (GDLs), and PTFE gaskets were obtained from Fuel Cell Store. Fumion® anion exchange resin was obtained from Fumatech BWT GmbH, Germany. PtRu/C catalyst (40% Pt and 20% Ru on carbon black, HiSPEC 10000) from Alfa Aesar was used for the anodes. Vulcan XC-72 carbon black from CABOT Corporation, henceforth Vulcan carbon was used for the microporous layers on the cathode and to adjust hydrophobicity on the cathode. 50 wt% Nickel-Molybdenum on Ketjenblack, henceforth NiMo/KB was obtained from Pajarito Powder, USA. All the other chemicals were reagent grade and used as received unless otherwise stated.

Ethylene tetrafluoroethylene (ETFE) film (25 µm thickness, catalog number FP36-FM-000125) was purchased from Goodfellow (UK). Vinylbenzyl chloride monomer (VBC, 97%, a mixture of 3- and 4-isomers) was supplied by Sigma-Aldrich and used after the removal of inhibitors (50–100 ppm 4-tert-butylcatechol and 700–1100 ppm nitromethane) using an aluminum oxide column. 1-Octyl-2-pyrrolidone and aqueous trimethylamine solution (TMA, 45% wt) were also obtained from Sigma-Aldrich. All chemicals were used as received, and ultra-pure water (UPW) with a resistivity of 18.2 MΩ cm was used.

Preparation of Catalysts

A solution of 1,3,5-tris(4-aminophenyl)benzene (TAPB, 1.41 g, 4 mmol) in acetone (45 mL) was added to a solution of pyromellitic dianhydride (1.31 g, 6 mmol), $Fe(acac)_3$ (60.3 mg, 0.15 mmol) and *N,N*-dimethyldodecylamine (0.3 mL) in acetone. The mixture was stirred for 30 min at 0 °C. After evaporation of the solvent, the residue was heated at 240 °C under evacuation to obtain polyimide nanoparticles. This Fe-containing polyimide (PI) was heated at 900 °C for 5 h in a nitrogen atmosphere and at 800 and 1000 °C for 1 h each in an ammonia atmosphere (50% counter-balanced by nitrogen). The obtained sample was referred to as Fe(SA+Nano)/PI.

For the purification to prepare a single-atom catalyst, Fe(SA+Nano)/PI (400 mg) was dispersed in isopropanol (100 mL). A magnetic rod (Magtec, MNMB-BW10-15, 1000 mT) was placed in the dispersion for 1 h and then was removed for washing. After repeating the

immersion and washing cycle seven times, the magnetic rod was returned to the dispersion overnight, after which the dispersion was filtered. The obtained residue was dried under a flow of nitrogen at 200 °C and was referred to as Fe(SA)/PI.

Radiation Grafted Anion-Exchange Membrane (RG-AEM) Synthesis

The synthesis procedure was adapted from previously published papers, which have been extensively studied and reported in the literature.[1-4] The RG-AEM was prepared using the pre-irradation method. The film was irradiated in an electron-beam accelerator in Sor-Van Radiation Ltd., Israel) with 5 MeV maximum energy. The sample was exposed to a total absorbed dose of 100 kGy (25 kGy per pass) under air atmosphere at room temperature. After irradiation, the film was transported back to the laboratory in dry ice and stored in a freezer at −40 °C.

For the grafting step, the irradiated film (100 cm^2 in area) was immersed in an aqueous mixture containing VBC (5% v/v) and 1-octyl-2-pyrrolidone dispersant (1% v/v) in glass vessels. The grafting mixture was purged with N_2 for 1-h, followed by an additional 1-h with the film inside. Finally, the vessel was completely sealed and heated at 70 °C for 16-h. After the grafting reactions were complete, the film was removed from the solution, washed with an excess of toluene, and dried in a desiccator overnight.

For the amination step, the ETFE-g-poly(VBC) intermediate membrane was immersed in an aqueous TMA solution at room temperature for 24-h. The resulting crude RG-AEM was thoroughly washed with UPW and then heated in UPW at 60 °C for 1-h. After amination, the membrane was converted to the Cl^- form by immersing it in a 1 M aqueous NaCl solution overnight, with the solution being replaced at least three times. The AEM was then soaked in UPW to remove excess of Na^+ and Cl^- ions and stored in fresh UPW until further use.

Materials Characterization

The CHN contents were determined by using a J-Science JM10 analyzer. The Fe content was determined by wavelength dispersive spectrometry (WDS) on an electron probe microanalyzer (EPMA; JXA-8100, Jeol). A LiFH crystal was used for Fe detection. The EPMA measurement was conducted by analyzing a pellet of the catalyst powder, which was prepared without any binder. The specific surface area of the catalysts was determined by N_2 adsorption using a volumetric adsorption measurement instrument (Bel Japan, Belsorp-mini II). Raman spectroscopy was performed using a JASCO RMP-510 spectrometer. Transmission electron microscopy (TEM) was conducted at 200 kV using a microscope (JEM-2010F, Jeol) equipped

with an energy-dispersive X-ray spectrometer (EDX, Genesis, EDAX). Scanning transmission electron microscopy (STEM) Was performed on a Thermo Scientific™ Talos F200 × 200 kV D6329 XTwin TEM. The samples were prepared by dispersing a small amount of the powder in 50%/50% vol water/n-propanol, sonicated for 15 min and drop-casted on a copper TEM grid. Images were collected in both Bright Field and High Angle Annular Dark Field (HAADF) Scanning Transmission Electron Microscopy (STEM) modes, while elemental spectra were collected using Energy Dispersive Spectroscopy (EDS) with ChemiSTEM™ technology and analyzed using ESPRIT Microanalysis Software. Additional HAADF-STEM images were acquired to show single atoms in the FE(SA)/PI catalyst using a JEOL-ARM200F at an accelerating voltage of 60 kV.[5]

Electrochemical Characterization

The catalytic activity of the materials towards alkaline ORR was tested by linear sweep voltammetry (LSV) at 10 mV sec^{-1} using an RRDE. The Fe-based catalysts (5 mg) were dispersed in a solution of water (150 μL), ethanol (150 μL), and Nafion DE521 (Aldrich, 50 μL), and 4 μL of the prepared ink was coated on the glassy carbon disk (0.283 cm^2) of an RRDE. The amount of the loaded catalyst was 60 μg cm^{-2}. The rotation speed of the RRDE was 1600 rpm with a Nikko Keisoku RDE-1 system, and the electrochemical data were collected with a potentiostat (HZ-7000, Hokuto Denko). The LSV was first carried out in an N_2-saturated 0.1 M KOH solution, and then in an O_2-saturated 0.1 M KOH solution. The ORR current was determined by subtracting the N_2 current from the O_2 current. The Pt ring current was recorded at 1.1 V vs. a reversible hydrogen electrode (RHE).

XAFS

Fe K-edge X-ray absorption fine structure (XAFS) spectra of Fe(SA)/PI and Fe(Nano+SA)/PI were taken on the NanoTerasu BL08W beamline. The spectra of α-Fe_2O_3 and Fe foil (0.01 mm thickness, The Nilako Corporation, Japan) were also measured as references. All spectra were measured in a transmission mode where monochromated X-ray with Si(111) double crystal were irradiated to a sample, and the incident and transmitted X-rays were detected using a 15 cm N_2 ion chamber and a 30 cm N_2 85%-Ar 15% ion chamber, respectively. The spectra were analyzed using Athena and Artemis software included in the Demeter package.

Characterization of AEMs

The degree of grafting (DoG, %) was calculated as follows:

$$DoG(\%) = 100 \times (m_g - m_i)/m_i$$

where m_g is the mass of the membrane after grafting, and m_i is the initial mass of the irradiated film. The ion exchange capacity (IEC) of the AEM was determined in Cl^- form using an automatic titrator (751 GPD Titrino, Metrohm) equipped with a selective Cl^- electrode following a procedure described elsewhere.[6]

AEMFC Characterization

The gas diffusion electrodes (GDEs) in this study were prepared by hand-spray-deposition of catalyst ink suspensions, as reported elsewhere,[7-9] and had active areas of 2.25 cm^{-2}. In brief, the catalyst ink suspensions for the cathode were made by combining ionomer and either the Fe(SA)/PI or Fe(Nano+SA)/PI into a mortar at a solid mass ratio of 0.425:1 (ionomer to catalyst). The ionomer was added first and ground for 3 min with a pestle to reduce the size of any agglomerated particles. Next the Fe(SA)/PI or Fe(Nano+SA)/PI were combined with the freshly ground ionomer in the mortar and deionized water (2 mL) with isopropanol (15 mL) to create catalyst ink suspensions. After grinding for approximately 10 min, the ink suspensions were transferred to 50 mL plastic centrifuge tubes and sonicated in an ice-cooled Elmasonic P 60 H ultrasonic bath at 180 W and 37 kHz for an hour. A sonicated suspension of Vulcan carbon in isopropanol was hand-sprayed deposited onto GDLs and dried on a hot plate at 140 ℃ to create GDLs with microporous layers (MPLs) of ~10 μm. The Fe(SA)/PI and Fe(Nano+SA)/PI catalyst ink suspensions were then hand-spray deposited onto the GDLs with MPLs, dried at 140 ℃, and weighed periodically until the catalyst loadings reached 1.5 ± 0.05 $mg_{Total\ catalyst}\ cm^{-2}$.

The PtRu/C anodes were prepared similarly with a solid mass ratio of 0.625:1.5:1 (ionomer to carbon to catalyst). First, the appropriate amount of ionomer was added to the mortar and ground, followed by the PtRu/C catalyst, extra Vulcan carbon, and solvents. The sonicated ink suspension was hand-spray deposited onto bare GDLs without MPLs to a catalyst loading of 0.6 $mg_{PtRu}\ cm^{-2}$. Additional, lower PGM loading anodes of 0.2 and 0.1 $mg_{PtRu}\ cm^{-2}$ were made in the same way with the addition of MPLs under the catalyst layers of ~20 μm thick. The NiMo/KB anodes had an ionomer to total catalyst ratio of 1:4 and were sprayed onto bare GDLs without MPLs to a catalyst loading of 13 $mg_{NiMo}\ cm^{-2}$.

To convert the AEM and ionomer contained in the catalyst layers of the GDEs to their hydroxide forms before each AEMFC test, a 5 cm^2 piece of AEM (100 μm average fully hydrated thickness) and the GDEs were placed to soak in individual Petri dishes of an aqueous

1 M KOH solution for an hour, with the solution refreshed every 20 min. Following the soaking, the cathodes, AEM, and PtRu/C anodes were stacked within PTFE gaskets with an average cell compression of 35% to prepare the membrane electrode assemblies (MEAs).

The individual MEAs were placed between a pair of 5 cm^2 single-serpentine graphite bipolar flow field plates and the 8 bolts were torqued to a value of 4.5 N m. Tests were performed in an 850E Scribner Associates Fuel Cell test station with $H_2 - O_2$ gas flows of 1 slpm at operating temperatures of 60 ºC without back-pressurization, and 80 ºC with back-pressurization of 200 kPag. For optimal performance, the anode and cathode dew point temperatures—which are indicated in the figure captions—were adjusted to balance the water inside the cell.[10-12] Polarization curves were obtained by scanning from OCV to ~0.3 V with voltage increments of 50 mV, stabilized at each point for 5 sec. Following the polarization scans, short-term AEMFC durability tests were conducted on the 0.6 and 0.2 mg_{PtRu} cm^{-2} anode AEMFCs by holding the current density constant at 1000 and 600 mA cm^{-2}, respectively, and reducing the H_2-O_2 gas flow rates to 0.2 slpm and back-pressurization to 150 kPag with all other operating conditions maintained.

For the fully CRM-free cells, the PtRu/C anode was switched to a NiMo/KB anode with a loading of 13 mg_{NiMo} cm^{-2}. The cell was initially operated at 80 °C with 250 kPag of back-pressurization and dewpoints opttimized to 78 and 80 °C on anode and cathode with $H_2 - O_2$ gas flows of 1 slpm. For the HT-AEMFC, the gas flow rates and back-pressure settings were maintained, while the cell temperature was increased to 118 °C and anode and cathode dewpoints were optimized to 116 and 117 °C, respectively. Polarization curves in O_2 mode were obtained by scanning from OCV to ~1.2 A with current steps of 25 mA, held each point for 5 sec. The oxidant was then switched to ambient air with all other operating conditions maintained and polarization curves by scanning from OCV to ~0.9 A with current steps of 25 mA, held each point for 5 sec. Following the polarization scans, a short-term HT-AEMFC durability test was conducted under a current density load of 250 mA cm^{-2} and reduced H_2-ambient air gas flow rates of 0.2 slpm, with all other settings unchanged.

Computational details

All theoretical calculations were performed by density functional theory (DFT), as implemented in the Vienna Ab-initio Simulation Package (VASP) code.[13] The electron-ion interactions were treated using the projector augmented wave (PAW) method,[14] while the Perdew-Burke-Ernzerhof (PBE) functional within the generalized gradient approximation (GGA) was employed to describe the exchange-correlation energy.[15] A cutoff energy of 400

eV was applied, with convergence thresholds set to 1×10^{-5} eV for total energy and 0.01 eV/Å for forces. A Monkhorst-Pack k-point grid of 3×3×1 was used to sample the Brillouin zone.[16]

The free energy diagrams of the oxygen reduction reactions (ORR) have been calculated according to the method developed by Nørskov *et al.*[17] For each elementary step, the Gibbs reaction free energy ΔG is defined as the difference between free energies of the initial state and final state and is calculated using the following equation:

$$\Delta G = \Delta E + \Delta ZPE - T\Delta S + eU \quad (1)$$

where ΔE is the binding energy of adsorption species OOH^*, O^*, and OH^*, ΔZPE is the change in zero-point energy, T is the temperature (298.15 K), and ΔS is the change in entropy, U is applied potentials. The binding energy of adsorption species OOH^*, O^*, and OH^* was calculated using the following equation:

$$\Delta E_{OH^*} = E_{OH^*} - E_* - (E_{H_2O} - \frac{1}{2}E_{H_2}) \quad (2)$$

$$\Delta E_{OOH^*} = E_{OOH^*} - E_* - (2E_{H_2O} - \frac{3}{2}E_{H_2}) \quad (3)$$

$$\Delta E_{O^*} = E_{O^*} - E_* - (E_{H_2O} - E_{H_2}) \quad (4)$$

where E_*, E_{OOH^*}, E_{O^*}, and E_{OH^*} are the ground state energies of the clean surface and the surfaces with OOH^*, O^*, and OH^* adsorbed, respectively. E_{H_2O}, E_{H_2} are the calculated DFT energies of H_2O and H_2 molecules in the gas phase.

1D AEMFC model simulation

To gain further insights into the enhanced durability of our Fe(SA)/PI cathode cell compared to the Fe(SA+Nano)/PI cathode cell, we employed our 1D AEMFC model.[18-19] The computational domain comprises a 5-layer MEA with a membrane, anode and cathode catalyst layers (CLs), and anode and cathode GDLs. The model takes multiple phenomena into account, including gas, water and ion transport as well as the electrochemical reactions in the CLs; hydrogen oxidation reaction (HOR) in the anode and oxygen reduction reaction (ORR) in the cathode. We additionally consider the degradation kinetics of the ionomeric materials in the membrane and the anode and cathode CLs. Our model offers a thorough understanding of the changes taking place in the cell over time by on account of each of these variables.

The molar concentrations of hydrogen, oxygen, nitrogen, and water, along with molar velocities, partial pressures, and electric fields, are the main dependent variables in our model. Water is modeled as three different forms: liquid, vapor, and within the ionomer, while liquid water in the CL impacts the porosity. While transport via the GDL is single-phase and studied using a Maxwell–Stefan approach with Darcy's law and water transport across the membrane

is represented as a combination of diffusion and electro-osmotic drag. The kinetics of the HOR and ORR are realized by modeling the electrochemical rea reactions using Butler–Volmer kinetics.

Supplementary Tables

Table S1. EXAFS curve-fitting result for Fe(SA)/PI.[a)]

CN(Fe-N) [b)]	*R* [c)] / Å	σ^2 [d)] / $Å^2$	E_0 [e)] / eV	R-factor
3.7(10)	2.01(3)	0.0069(37)	7109.9(41)	0.0034

[a)] FT range: 3–14 $Å^{-1}$, curve-fitting range: 1.1–2.0 Å. S_0^2 was assumed to be 1. [b)] Coordination number of Fe-N. [c)] Atomic distance. [d)] Debye–Waller factor. [e)] Absorption edge energy.

Table S2. Performance comparison of single atom metal-based PGM-free cathode catalysts in AEMFCs. All reports herein are from the past 5 years containing PGM-based anodes and operated with $H_2|O_2$.

Cathode catalyst | loading [mg cm^{-2}]	Anode catalyst | PGM loading [mg cm^{-2}]	Cell Temp [ºC]	Current density @ 0.80 V [A cm^{-2}]	Current density @ 0.65 V [A cm^{-2}]	P_{max} [W cm^{-2}]	Specific power [W mg_{PGM}^{-1}]	Durability test current density | Final time [mA cm^{-2}] | [h]	Durability test voltage degradation rate | % [mV h^{-1}] | [%]	Ref
Fe(SA)/PI | 1.5	PtRu | 0.6	80	0.96	2.73	1.79	2.98	1000 | 50	2 | 18	**This work**
Fe(SA)/PI | 1.5	PtRu | 0.2	80	0.62	2.36	1.79	8.95	600 | 100	0.8 | 7	**This work**
Fe(SA)/PI | 1.5	PtRu | 0.1	80	0.57	1.58	1.13	11.3	-	-	**This work**
Fe(SA+Nano)/PI | 1.5	PtRu | 0.6	80	0.41	2.02	1.51	2.52	1000 | 50	6.9 | 54	**This work**
Fe-N-C | 1.0	PtRu | 0.6	80	0.57	2.10	2.05	3.42	600 | 150	0.46 | 10	[20]
Fe-N-C | 1.0	PtRu | 0.125	80	0.30	1.32	1.30	10.4	1000 | 3.5	-	[20]
Fe-Mn-N-C | 0.8	PtRu | 0.4	80	0.34	1.30	1.32	3.3	400 | 50 [a)]	7.2 | 59 [a)]	[21]
FeCoNC-MgOAc | 0.96	PtRu | 0.57	60	0.20	0.94	1.12	1.96	600 | 24	1.7 | 5	[22]
$Fe_{0.5}$-dry | 0.91	PtRu | 0.6	80	0.67	1.99	1.80	3	-	-	[23]
$Fe_{0.5}$-dry | 0.91	PtRu | 0.135	80	0.36	2.03	1.10	8.15	-	-	[23]
CoFe-N-CDC/CNT | 0.75	PtRu | 0.74	60	0.16	1.24	1.12	1.51	600 | 20	1.5 | 9	[24]
FeNC-20 | 2.0	PtRu | 0.4	60	0.15	0.65	0.59	1.58	-	-	[25]
MPF/Fe | 1.0	PtRu | 0.4	65	0.13	0.40	0.35	0.88	-	-	[26]
L_FeMn | 0.8	PtRu | 0.4	60	0.06	0.26	0.26	0.65	-	-	[27]
Ag_{NPs}@Fe-N-C | 2.0	PtRu | 0.4	80	0.18	0.76	0.85	2.13	-	-	[28]

$Fe_{0.5}$-NH_3 | 1.5	PtRu | 0.9	60	0.35	1.10	1.04	1.16	-	-	[29]
FeCu-NC | 1	PtRu | 0.4	80	0.35	1.24	0.91	2.28	400 | 30	12.7 | 63	[30]
Fe-NC | 1	PtRu | 0.4	80	0.27	1.0	0.76	1.9	-	-	[30]
Co_1/CNH 700 | 6	PtRu | 0.6	60	0.15	0.52	0.47	0.79	-	-	[31]
C@PVI-(DFTPP)Fe-800 | 2	Pt | 2	60	0.01	0.08	0.10	0.05	-	-	[32]
HT800-FeP-aerogel | 1.25	PtRu | 0.71	80	0.05	0.37	0.58	0.82	-	-	[9]
CoTPyP@Im-RGO | 3	PtRu | 0.3	80	0.11	0.34	0.53	1.76	-	-	[33]
Co_{NP}/$CNH_{3.6\%}$ NH_3 | 1	Pt | 0.4	60	0.02	0.5	0.62	1.55	-	-	[34]
Co_{NP}/$CNH_{8.1\%}$ NH_3 | 1	Pt | 0.4	60	0.07	0.93	0.75	1.86	-	-	[34]
Co/CNH Air NH_3 | 2	PtRu | 0.6	60	0.1	0.64	0.74	1.23	-	-	[35]
ZnNC | 1	PtRu | 0.6	80	0.46	1.59	1.63	2.72	-	-	[36]
ZnNC | 1	PtRu | 0.2	80	0.4	1.37	1.33	6.65	400 | 100 [b)]	2.3 | 30 [b)]	[36]
BMOF-derived Zn_{20}Co | 0.5	PtRu | 0.4	80	0.13	0.87	0.93	2.33	200 | 24	6 | 20.7	[37]
Fe-N-C-2 | 5	Pt | 0.4	55	-	0.14	0.15	0.38	-	-	[38]
Cu–N/B–C-800 | 2	Pt | 0.4	60	0	0.05	0.08	0.2	-	-	[39]
O-FeN_4C-O | 0.8	PtRu | 0.4	80	0.37	1.15	1.24	3.1	-	-	[40]
SA-Fe/NG | 0.8	PtRu | 0.4	80	0. 18	0.92	0.99	2.28	50 | 10	49.2 | 51	[41]

[a)] Fe-Mn-N-C loading for the durability test was 1 $mg_{Fe-Mn-N-C}$ cm^{-2}. [b)] PtRu loading for the durability test was 0.4 mg_{PtRu} cm^{-2}.

Table S3. Performance comparison of our critical raw material (CRM)-free catalyst AEMFCs against other PGM- and CRM-free catalyst AEMFCs reported in the literature operated with $H_2|O_2$.

Cathode catalyst | loading [mg cm^{-2}]	Anode catalyst | PGM loading [mg cm^{-2}]	Cell Temp [ºC]	Current density @ 0.80 V [mA cm^{-2}]	Current density @ 0.65 V [mA cm^{-2}]	P_{max} [mW cm^{-2}]	P_{max} current density [mA cm^{-2}]	Ref
Fe(SA)/PI [a)] | 1.5	NiMo/KB [a)] | 13	118	53	298	372	998	**This work**
Fe(SA)/PI [a)] | 1.5	NiMo/KB [a)] | 13	80	26	97	135	345	**This work**
N-doped-C [a)] | 0.7	Ni_7Fe/C [a)] | 3	95	32	63	55	137	[42]
$MnCo_2O_4$/C | 0.2	Ni-H_2-NH_3 [a)] | 6	95	226	601	488	919	[43]
$MnCo_2O_4$ | 1.5	Ni@CN_x [a)] | 15	80	117	275	210	450	[44]
V-AgNs | 0.25	Ni/C [a)] | 1.25	60	0	4	115	405	[45]
S-AgNs | 0.25	Ni/C [a)] | 1.25	60	0	0	41	292	[45]
Ag | 1	Cr-decorated Ni [a)] | 5	60	13	65	50	97	[46]
CoPPY/C [a)] | 2	Ni-W [a)] | 17.5	60	4	28	40	104	[47]
Ag | 0.5	Ni/C | 5	70	67	113	76	113	[48]
Co_3O_4/C [a)] | 3	NiCo/C [a)] | 5	70	0. 6	13	22	34	[49]
ZrN | 2	Ni_3N | 4	80	54	115	161	540	[50]
ZrN | 2	Ni_3N | 4	90	54	228	256	642	[50]
Ag/C | 0.6	NiCuCr/C | 9	80	71	334	335	762	[51]

[a)] Critical raw material (CRM)-free catalyst used.

Table S4. Performance comparison of our critical raw material (CRM)-free catalyst AEMFC against the only other CRM-free catalyst AEMFCs reported in the literature operated with H_2|ambient air.

Cathode catalyst | loading [mg cm^{-2}]	Anode catalyst | PGM loading [mg cm^{-2}]	Cell Temp [ºC]	Current density @ 0.80 V [A cm^{-2}]	Current density @ 0.65 V [A cm^{-2}]	P_{max} [W cm^{-2}]	P_{max} current density [mA cm^{-2}]	Durability test current density | Final time [mA cm^{-2}] | [h]	Durability test voltage degradation rate | % [mV h^{-1}] | [%]	Ref
Fe(SA)/PI | 1.5	NiMo/KB | 13	118	8	98	206	652	225 | 5	10 | 10	**This work**
CoPPY/C | 2	Ni-W | 17.5	60	0.2	22	27.5	65	-	-	[47]

Supplementary Figures

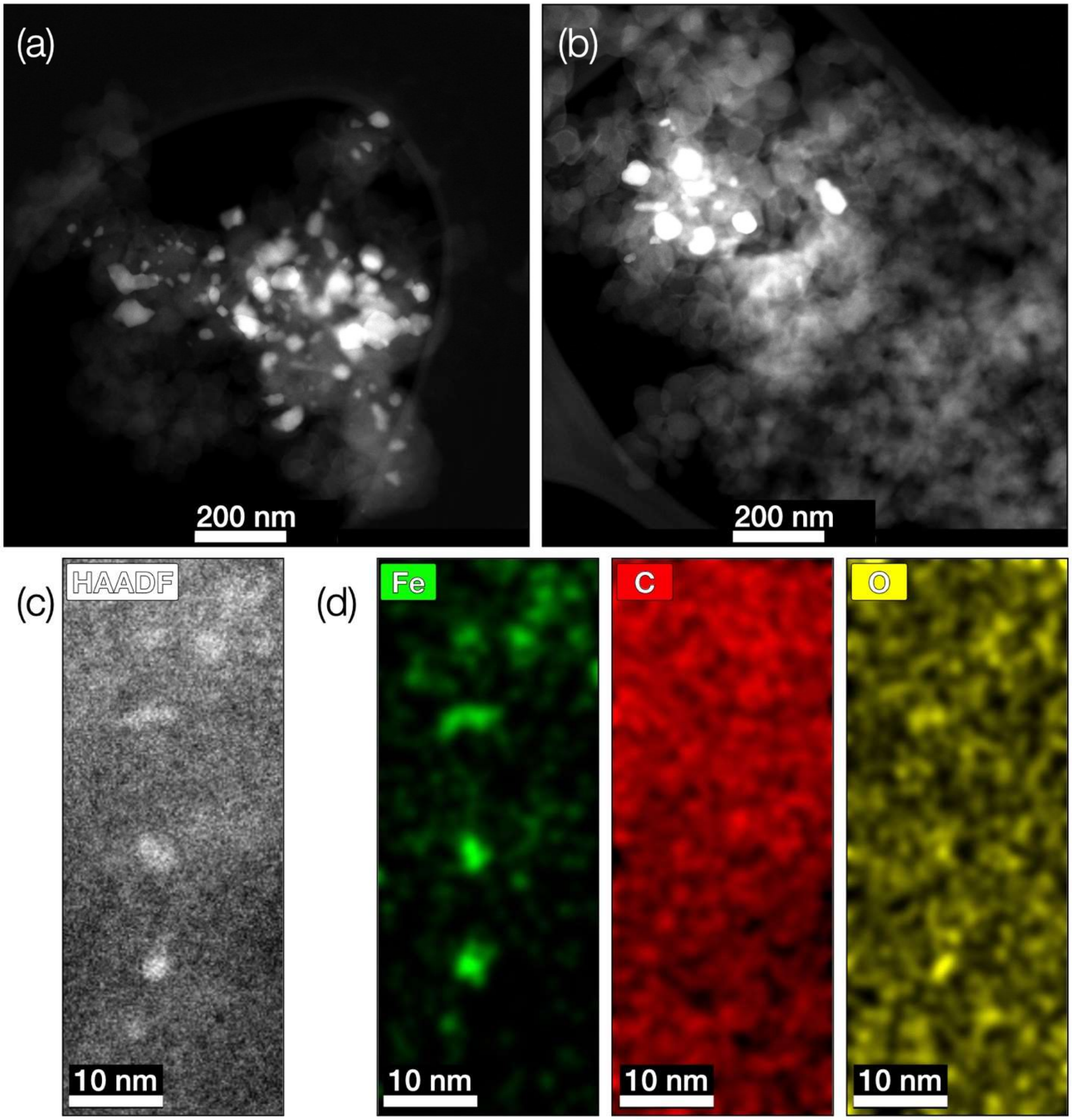

Figure S1. Scanning transmission electron microscopy (STEM) images of (a) non-purified Fe(SA+Nano)/PI and (b) magnetically purified Fe(SA)/PI catalysts. (c) Layered HAADF-STEM image and (d) individual EDX elemental maps showing Fe, C and O in the Fe(SA)/PI catalyst. Although some forms of iron clusters are exhibited in the catalyst layer as shown in (c) and (d), they can be attributed to sample preparation for imaging.

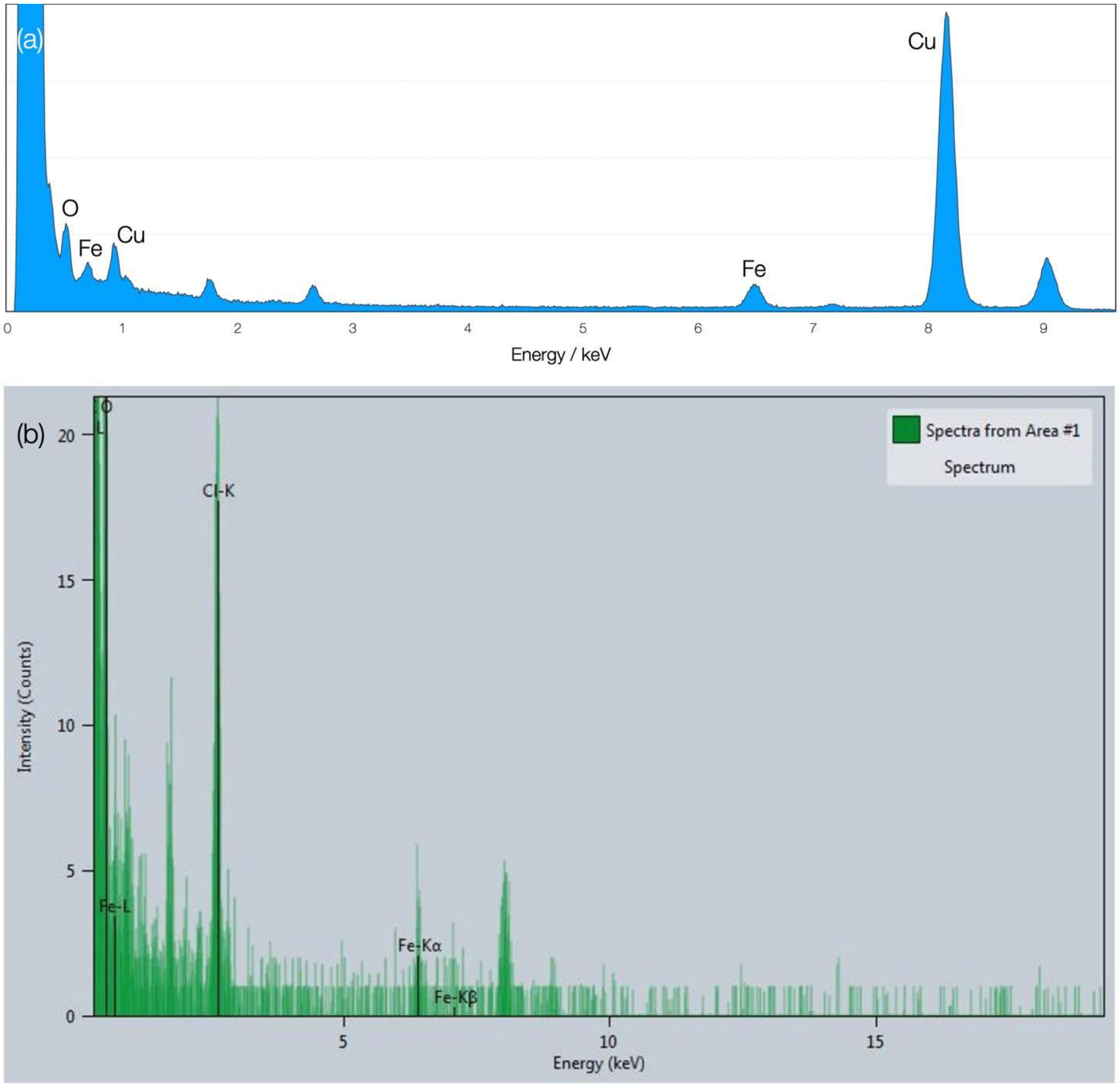

Figure S2. EDX elemental spectrum recorded in (a) Figure 1(e), and (b) Figure S1(c), respectively.

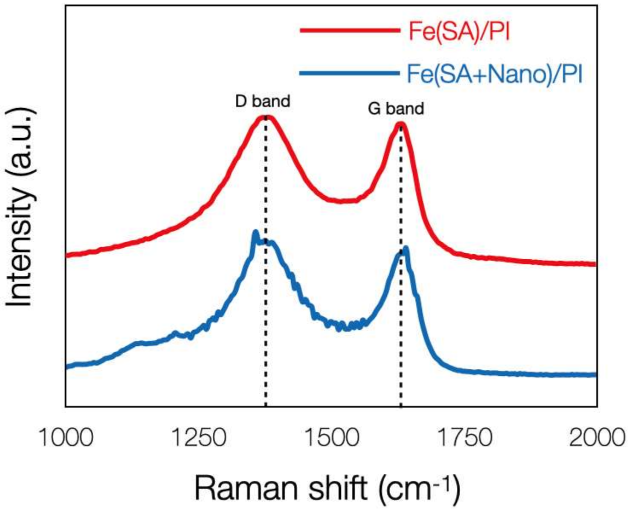

Figure S3. Raman patterns of Fe(SA)/PI and Fe(SA+Nano)/PI catalysts.

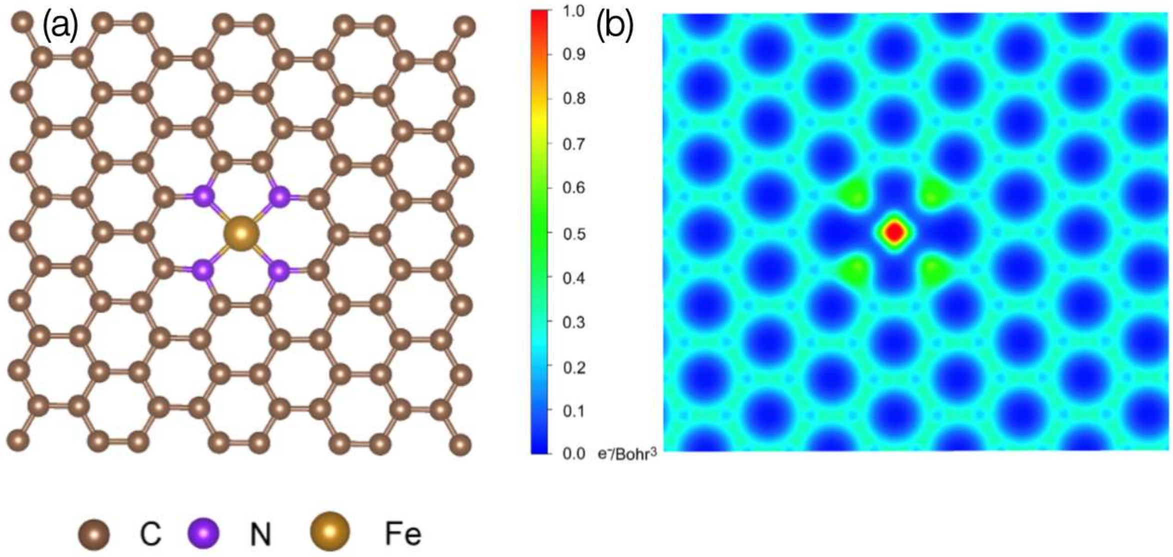

Figure S4. (a) Optimized geometric model and (b) charge density distribution of FeN_4.

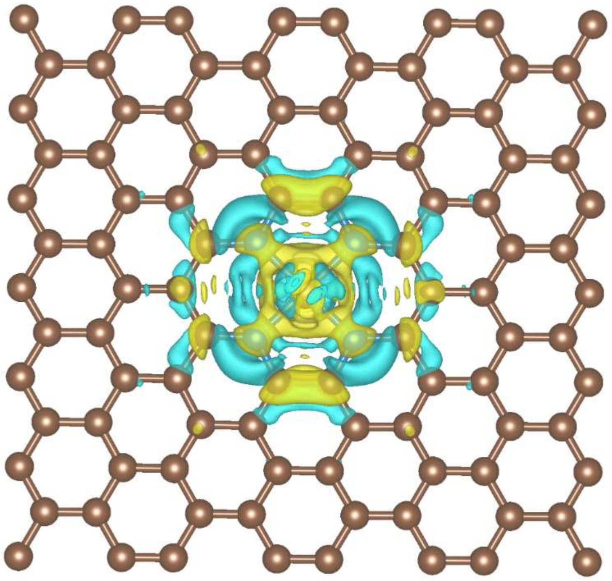

Figure S5. The charge density difference map of Fe$\mathbf{N_4}$, yellow indicates electron accumulation and cyan represents electron depletion, with an isosurface value of 0.002 e $Å^{-3}$.

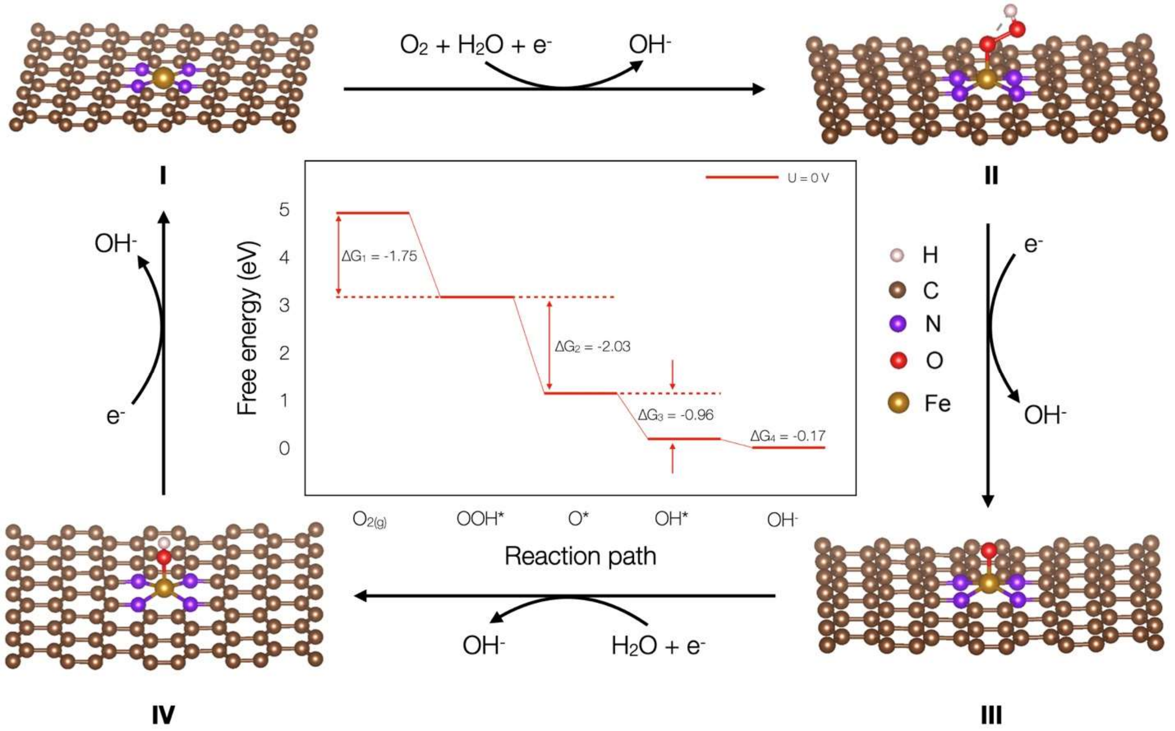

Figure S6. The ORR reaction pathway and free energy diagram Fe(SA)/PI in alkaline media.

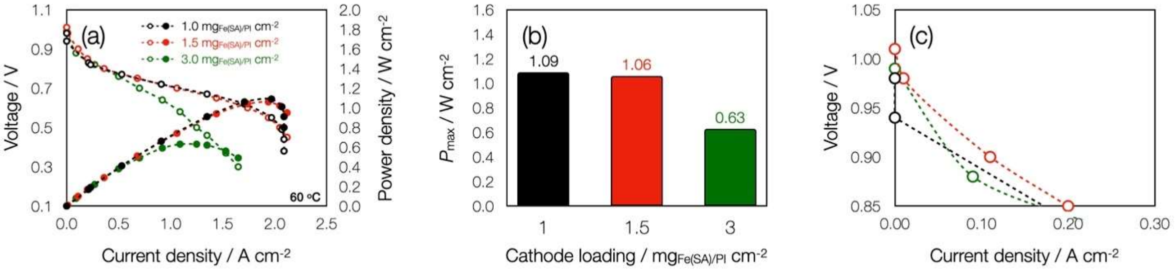

Figure S7. AEMFCs operated with $H_2|O_2$ at 60 ℃ without back-pressurization and anode and cathode dewpoints of 54 and 56 ℃, respectively used to determine ideal catalyst loading for all experiments showing (a) polarization curves (V, empty symbols, Y1 axis) and power density curves (filled symbols, Y2 axis) as a function of current density, (b) peak power density comparison with different loadings and (c) a zoom-in of the kinetic regions. AEMFCs were synthesized with Fe(SA)/PI cathode catalysts loadings of 1, 1.5, and 3 $mg_{Fe(SA)/PI}$ cm^{-2}, PtRu/C anodes containing 20 wt% ionomer loaded to 0.6 mg_{PtRu} cm^{-2} and ETFE-based AEMs.

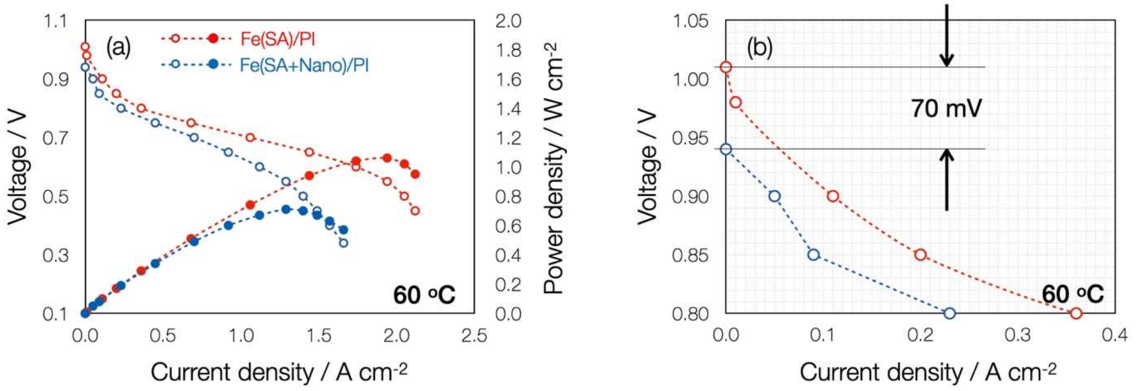

Figure S8. $H_2|O_2$ polarization curves (V, empty symbols, Y1 axis) and power density curves (filled symbols, Y2 axis) as a function of current density for AEMFCs with Fe(SA)/PI and Fe(SA+Nano)/PI cathode catalysts with the same loadings of 1.5 $mg_{Total\ catalyst}$ cm^{-2} showing (a) full curves and (b) a zoom-in of the kinetic regions. The AEMFCs were operated with $H_2|O_2$ at 60 ℃ without back-pressurization and anode and cathode dewpoints of 54 and 56 ℃, while having PtRu/C anodes containing 20 wt% ionomer loaded to 0.6 mg_{PtRu} cm^{-2} and ETFE-based AEMs.

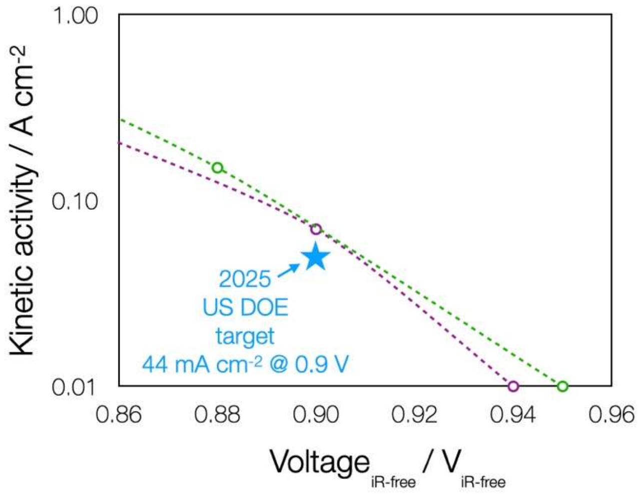

Figure S9. Determination of the catalytic activities in $H_2|O_2$ at 0.9 $V_{iR\text{-}free}$ using the cells from Figure 5(b) at 80 ºC with 100 kPag of back-pressurization and full humidification. The blue star denotes the US DOE target for 2025.[52]

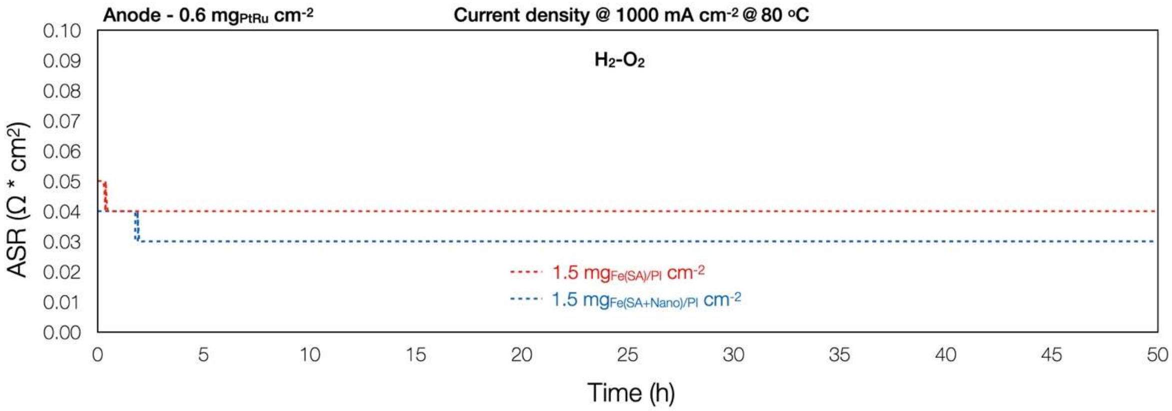

Figure S10. Area specific resistance (ASR) plots from the 50-h durability tests of Fe(SA)/PI and Fe(SA+Nano)/PI cathodes at a constant current density of 1000 mA cm^{-2} shown in Figure 5(c). Both cells had PtRu/C anodes with 0.6 mg_{PtRu} cm^{-2} with the cathode loadings were 1.5 $mg_{Total\ catalyst}$ cm^{-2}, and test conditions were 80 ºC with 150 kPag of back-pressurization and dewpoints set at 74 and 76 ℃ on the anode and cathode, respectively.

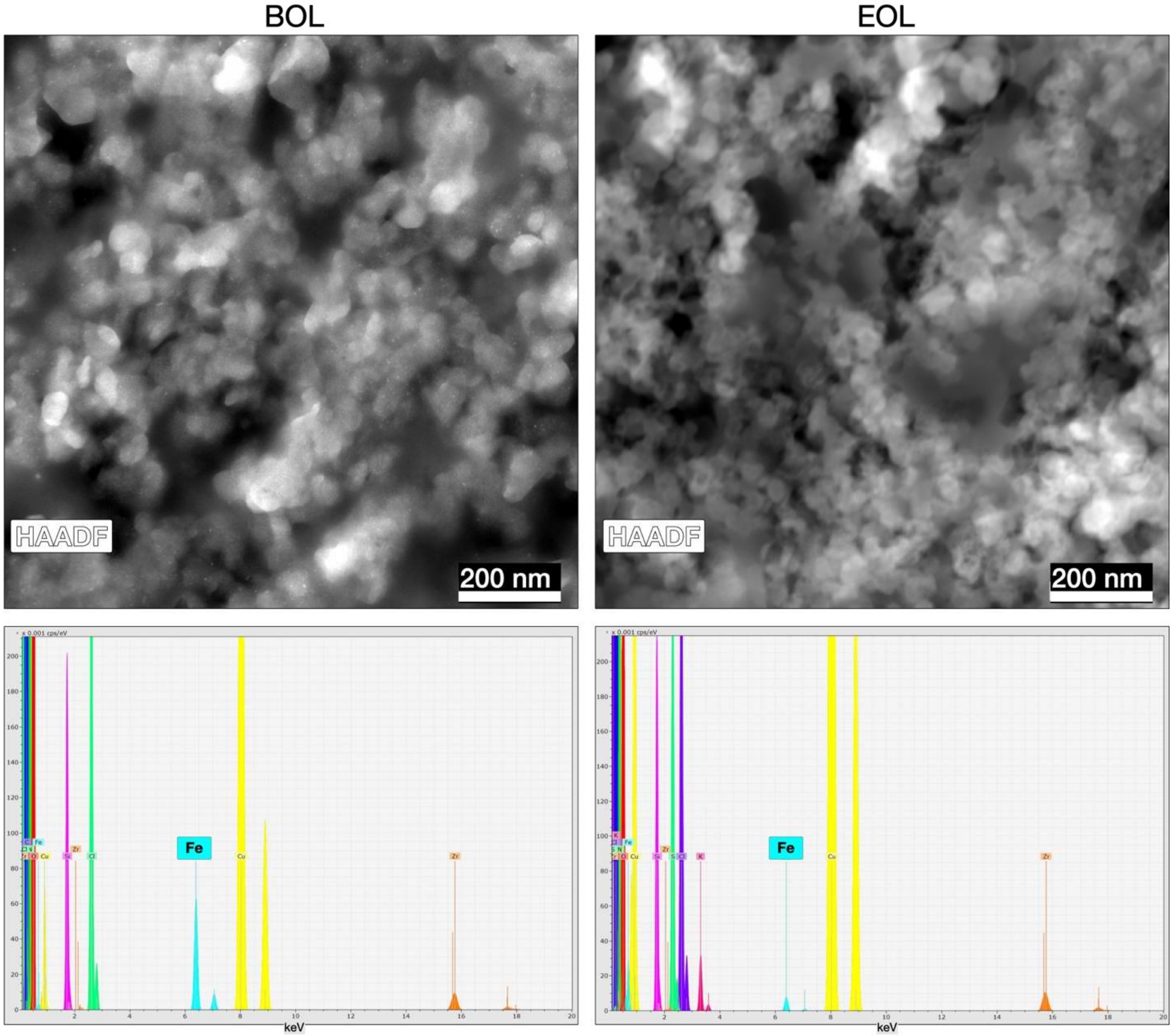

Figure S11. Layered HAADF-STEM and EDX spectra of BOF (left images) and EOL (right images) of magnetically purified Fe(SA)/PI catalyst layers before and after the durability testing at 1000 mA cm^{-2} from Figure 5(c).

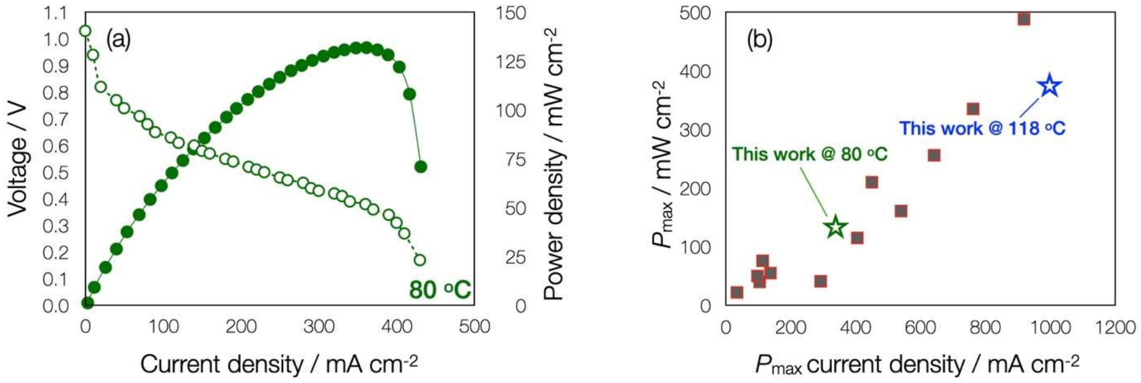

Figure S12. (a) $H_2|O_2$ polarization curves (V, empty symbols, Y1 axis) and power density curves (filled symbols, Y2 axis) as a function of the current density of a completely CRM-free AEMFC made with a 1.5 $mg_{Fe(SA)/PI}$ cm^{-2} cathode and NiMo/KB anode loaded to 13 mg_{NiMo} cm^{-2}, operated at 80 °C with 250 kPag of back-pressurization and dewpoints of 78 and 80 °C on anode and cathode flowing $H_2|O_2$. (b) Performance comparison of our completely CRM-free AEMFCs against other PGM-free and CRM-free catalyst AEMFCs reported in the literature[42-51] operated with $H_2|O_2$, comparing P_{max} values and the current density at which the P_{max} value was obtained.

References

[1] L. Wang, J. J. Brink, J. R. Varcoe, *Chem. Commun.* **2017**, *53* (86), 11771, 10.1039/c7cc06392j.
[2] A. L. G. Biancolli, S. Bsoul-Haj, J. C. Douglin, A. S. Barbosa, R. R. de Sousa, O. Rodrigues, A. J. C. Lanfredi, D. R. Dekel, E. I. Santiago, *Journal of Membrane Science* **2022**, *641*, 10.1016/j.memsci.2021.119879.
[3] S. Willdorf-Cohen, A. Zhegur-Khais, J. Ponce-González, S. Bsoul-Haj, J. R. Varcoe, C. E. Diesendruck, D. R. Dekel, *ACS Applied Energy Materials* **2023**, *6* (2), 1085, 10.1021/acsaem.2c03689.
[4] A. L. G. Biancolli, B. Chen, A. S. Menandro, F. C. Fonseca, E. I. Santiago, S. Holdcroft, *Journal of Materials Chemistry A* **2024**, 10.1039/d4ta02354d.
[5] H. Huang, Z. Zhang, C. Xiao, J. Liu, Z. Li, Y. Jiang, L. Wei, T. Zhao, F. Ciucci, L. Zeng, *Advanced Science* **2025**, 10.1002/advs.202505304.
[6] S. Haj-Bsoul, J. R. Varcoe, D. R. Dekel, *Journal of Electroanalytical Chemistry* **2022**, *908*, 10.1016/j.jelechem.2022.116112.
[7] J. C. Douglin, R. K. Singh, S. Haj-Bsoul, S. Li, J. Biemolt, N. Yan, J. R. Varcoe, G. Rothenberg, D. R. Dekel, *Chemical Engineering Journal Advances* **2021**, *8*, 10.1016/j.ceja.2021.100153.
[8] J. C. Douglin, R. K. Singh, E. R. Hamo, M. B. Hassine, P. J. Ferreira, B. A. Rosen, H. A. Miller, G. Rothenberg, D. R. Dekel, *Journal of Solid State Electrochemistry* **2022**, *26* (9), 2049, 10.1007/s10008-022-05261-4.
[9] N. Zion, J. C. Douglin, D. A. Cullen, P. Zelenay, D. R. Dekel, L. Elbaz, *Advanced Functional Materials* **2021**, *31* (24), 10.1002/adfm.202100963.
[10] R. Gutru, Z. Turtayeva, F. Xu, G. Maranzana, B. Vigolo, A. Desforges, *International Journal of Hydrogen Energy* **2020**, *45* (38), 19642, 10.1016/j.ijhydene.2020.05.026.
[11] M. M. Hossen, M. S. Hasan, M. R. I. Sardar, J. b. Haider, Mottakin, K. Tammeveski, P. Atanassov, *Applied Catalysis B: Environmental* **2023**, *325*, 10.1016/j.apcatb.2022.121733.
[12] T. J. Omasta, A. M. Park, J. M. LaManna, Y. Zhang, X. Peng, L. Wang, D. L. Jacobson, J. R. Varcoe, D. S. Hussey, B. S. Pivovar, W. E. Mustain, *Energy & Environmental Science* **2018**, *11* (3), 551, 10.1039/c8ee00122g.
[13] G. Kresse, J. Hafner, *Phys Rev B* **1993**, *48* (17), 13115, DOI 10.1103/PhysRevB.48.13115.
[14] G. Kresse, D. Joubert, *Phys Rev B* **1999**, *59* (3), 1758, DOI 10.1103/PhysRevB.59.1758.
[15] J. P. Perdew, K. Burke, M. Ernzerhof, *Phys Rev Lett* **1996**, *77* (18), 3865, DOI 10.1103/PhysRevLett.77.3865.
[16] H. J. Monkhorst, J. D. Pack, *Phys Rev B* **1976**, *13* (12), 5188, DOI 10.1103/PhysRevB.13.5188.
[17] J. K. Nørskov, J. Rossmeisl, A. Logadottir, L. Lindqvist, J. R. Kitchin, T. Bligaard, H. Jónsson, *The Journal of Physical Chemistry B* **2004**, *108* (46), 17886, 10.1021/jp047349j.
[18] D. R. Dekel, I. G. Rasin, S. Brandon, *Journal of Power Sources* **2019**, *420*, 118, 10.1016/j.jpowsour.2019.02.069.
[19] K. Yassin, J. C. Douglin, I. G. Rasin, P. G. Santori, B. Eriksson, N. Bibent, F. Jaouen, S. Brandon, D. R. Dekel, *Energy Conversion and Management* **2022**, *270*, 10.1016/j.enconman.2022.116203.
[20] H. Adabi, A. Shakouri, N. Ul Hassan, J. R. Varcoe, B. Zulevi, A. Serov, J. R. Regalbuto, W. E. Mustain, *Nature Energy* **2021**, *6* (8), 834, 10.1038/s41560-021-00878-7.
[21] S. Huang, Z. Qiao, P. Sun, K. Qiao, K. Pei, L. Yang, H. Xu, S. Wang, Y. Huang, Y. Yan, D. Cao, *Applied Catalysis B: Environmental* **2022**, *317*, 10.1016/j.apcatb.2022.121770.

[22] K. Kisand, A. Sarapuu, J. C. Douglin, A. Kikas, A. Treshchalov, M. Käärik, H.-M. Piirsoo, P. Paiste, J. Aruväli, J. Leis, V. Kisand, A. Tamm, D. R. Dekel, K. Tammeveski, *ACS Catalysis* **2022**, *12* (22), 14050, 10.1021/acscatal.2c03683.
[23] H. Adabi, P. G. Santori, A. Shakouri, X. Peng, K. Yassin, I. G. Rasin, S. Brandon, D. R. Dekel, N. U. Hassan, M.-T. Sougrati, A. Zitolo, J. R. Varcoe, J. R. Regalbuto, F. Jaouen, W. E. Mustain, *Materials Today Advances* **2021**, *12*, 10.1016/j.mtadv.2021.100179.
[24] J. Lilloja, E. Kibena-Põldsepp, A. Sarapuu, J. C. Douglin, M. Käärik, J. Kozlova, P. Paiste, A. Kikas, J. Aruväli, J. Leis, V. Sammelselg, D. R. Dekel, K. Tammeveski, *ACS Catalysis* **2021**, *11* (4), 1920, 10.1021/acscatal.0c03511.
[25] H.-W. Jang, G.-S. Kang, J. Y. Lee, S. Y. Lee, G. Lee, S. J. Yoo, S. Lee, H.-I. Joh, *Chemical Engineering Journal* **2023**, *474*, 10.1016/j.cej.2023.145464.
[26] S. Akula, M. Mooste, J. Kozlova, M. Käärik, A. Treshchalov, A. Kikas, V. Kisand, J. Aruväli, P. Paiste, A. Tamm, J. Leis, K. Tammeveski, *Chemical Engineering Journal* **2023**, *458*, 10.1016/j.cej.2023.141468.
[27] M. Muhyuddin, A. Friedman, F. Poli, E. Petri, H. Honig, F. Basile, A. Fasolini, R. Lorenzi, E. Berretti, M. Bellini, A. Lavacchi, L. Elbaz, C. Santoro, F. Soavi, *Journal of Power Sources* **2023**, *556*, 10.1016/j.jpowsour.2022.232416.
[28] Y. Yang, X. Xu, P. Sun, H. Xu, L. Yang, X. Zeng, Y. Huang, S. Wang, D. Cao, *Nano Energy* **2022**, *100*, 10.1016/j.nanoen.2022.107466.
[29] P. G. Santori, F. D. Speck, S. Cherevko, H. A. Firouzjaie, X. Peng, W. E. Mustain, F. Jaouen, *Journal of The Electrochemical Society* **2020**, *167* (13), 10.1149/1945-7111/abb7e0.
[30] Z. Xiao, P. Sun, Z. Qiao, K. Qiao, H. Xu, S. Wang, D. Cao, *Chemical Engineering Journal* **2022**, *446*, 10.1016/j.cej.2022.137112.
[31] J. Y. Jung, J. H. Jang, J. G. Kim, K. S. Lee, H. K. Lim, P. Kim, R. P. H. Chang, J. W. Park, S. J. Yoo, N. D. Kim, *Small Methods* **2021**, *5* (8), 10.1002/smtd.202100239.
[32] Y.-M. Zhao, P.-C. Zhang, C. Xu, X.-Y. Zhou, L.-M. Liao, P.-J. Wei, E. Liu, H. Chen, Q. He, J.-G. Liu, *ACS Applied Materials & Interfaces* **2020**, *12* (15), 17334, 10.1021/acsami.9b20711.
[33] B. Yang, X. Li, Q. Cheng, X. Jia, Y. Liu, Z. Xiang, *Nano Energy* **2022**, *101*, 10.1016/j.nanoen.2022.107565.
[34] J. Y. Jung, H. Jin, M. W. Kim, S. Kim, J.-G. Kim, P. Kim, Y.-E. Sung, S. J. Yoo, N. D. Kim, *Applied Catalysis B: Environmental* **2023**, *323*, 10.1016/j.apcatb.2022.122172.
[35] J. Y. Jung, S. Kim, J.-G. Kim, M. J. Kim, K.-S. Lee, Y.-E. Sung, P. Kim, S. J. Yoo, H.-K. Lim, N. D. Kim, *Nano Energy* **2022**, *97*, 10.1016/j.nanoen.2022.107206.
[36] P. Sun, Z. Qiao, S. Wang, D. Li, X. Liu, Q. Zhang, L. Zheng, Z. Zhuang, D. Cao, *Angewandte Chemie* **2022**, *135* (6), 10.1002/ange.202216041.
[37] W. Xu, R. Zeng, M. Rebarchik, A. Posada-Borbón, H. Li, C. J. Pollock, M. Mavrikakis, H. D. Abruña, *Journal of the American Chemical Society* **2024**, *146* (4), 2593, 10.1021/jacs.3c11355.
[38] Y. Jiang, H. Xu, B. Ma, Z. Zhang, Y. Zhou, *Fuel* **2024**, *366*, 10.1016/j.fuel.2024.131404.
[39] L. M. Liao, Y. M. Zhao, C. Xu, X. Y. Zhou, P. J. Wei, J. G. Liu, *ChemistrySelect* **2020**, *5* (12), 3647, 10.1002/slct.202000523.
[40] P. Sun, K. Qiao, D. Li, X. Liu, H. Liu, L. Yang, H. Xu, Z. Zhuang, Y. Yan, D. Cao, *Chem Catalysis* **2022**, *2* (10), 2750, 10.1016/j.checat.2022.09.009.
[41] L. Yang, H. Liu, Z. Qiao, P. Sun, D. Li, R. Jiang, S. Liu, Z. Niu, Y. Zhang, T. Lin, Q. Zhang, L. Gu, S. Wang, D. Cao, Z. Chen, *Advanced Energy Materials* **2023**, *13* (20), 10.1002/aenm.202204390.
[42] J. Biemolt, J. C. Douglin, R. K. Singh, E. S. Davydova, N. Yan, G. Rothenberg, D. R. Dekel, *Energy Technology* **2021**, *9* (4), 10.1002/ente.202000909.

[43] W. Ni, T. Wang, F. Héroguel, A. Krammer, S. Lee, L. Yao, A. Schüler, J. S. Luterbacher, Y. Yan, X. Hu, *Nature Materials* **2022**, *21* (7), 804, 10.1038/s41563-022-01221-5.
[44] Y. Gao, Y. Yang, R. Schimmenti, E. Murray, H. Peng, Y. Wang, C. Ge, W. Jiang, G. Wang, F. J. DiSalvo, D. A. Muller, M. Mavrikakis, L. Xiao, H. D. Abruña, L. Zhuang, *Proceedings of the National Academy of Sciences* **2022**, *119* (13), 10.1073/pnas.2119883119.
[45] P. Anandha Ganesh, A. N. Prakrthi, S. Selva Chandrasekaran, D. Jeyakumar, *RSC Advances* **2021**, *11* (40), 24872, 10.1039/d1ra02718b.
[46] S. Lu, J. Pan, A. Huang, L. Zhuang, J. Lu, *Proceedings of the National Academy of Sciences* **2008**, *105* (52), 20611, 10.1073/pnas.0810041106.
[47] Q. Hu, G. Li, J. Pan, L. Tan, J. Lu, L. Zhuang, *International Journal of Hydrogen Energy* **2013**, *38* (36), 16264, 10.1016/j.ijhydene.2013.09.125.
[48] S. Gu, W. Sheng, R. Cai, S. M. Alia, S. Song, K. O. Jensen, Y. Yan, *Chem. Commun.* **2013**, *49* (2), 131, 10.1039/c2cc34862d.
[49] V. Men Truong, J. Richard Tolchard, J. Svendby, M. Manikandan, H. A. Miller, S. Sunde, H. Yang, D. R. Dekel, A. Oyarce Barnett, *Energies* **2020**, *13* (3), 10.3390/en13030582.
[50] X.-L. Zhang, S.-J. Hu, Y.-H. Wang, L. Shi, Y. Yang, M.-R. Gao, *Nano Letters* **2022**, *23* (1), 107, 10.1021/acs.nanolett.2c03707.
[51] X. Wang, X. Liu, J. Fang, H. Wang, X. Liu, H. Wang, C. Chen, Y. Wang, X. Zhang, W. Zhu, Z. Zhuang, *Nature Communications* **2024**, *15* (1), 10.1038/s41467-024-45370-4.
[52] H. Zhang, L. Osmieri, J. H. Park, H. T. Chung, D. A. Cullen, K. C. Neyerlin, D. J. Myers, P. Zelenay, *Nature Catalysis* **2022**, *5* (5), 455, 10.1038/s41929-022-00778-3.
